# Mini-Puberty, Physiological and Disordered: Consequences, and Potential for Therapeutic Replacement

**DOI:** 10.1210/endrev/bnae003

**Published:** 2024-03-04

**Authors:** Julia Rohayem, Emma C Alexander, Sabine Heger, Anna Nordenström, Sasha R Howard

**Affiliations:** Department of Pediatric Endocrinology and Diabetology, Children’s Hospital of Eastern Switzerland, 9006 St. Gallen, Switzerland; University of Muenster, 48149 Muenster, Germany; Centre for Endocrinology, William Harvey Research Institute, Queen Mary University of London, London EC1M 6BQ, UK; Department of Pediatric Endocrinology, Children's Hospital Auf der Bult, 30173 Hannover, Germany; Pediatric Endocrinology, Karolinska Institutet, Astrid Lindgren Children's Hospital, Karolinska University Hospital, 17176 Stockholm, Sweden; Centre for Endocrinology, William Harvey Research Institute, Queen Mary University of London, London EC1M 6BQ, UK; Department of Paediatric Endocrinology, Royal London Children's Hospital, Barts Health NHS Trust, London E1 1FR, UK

**Keywords:** mini-puberty, hypogonadism, gonadotropins, puberty, infancy, cryptorchidism, Kallmann syndrome, congenital hypogonadotropic hypogonadism, micropenis

## Abstract

There are 3 physiological waves of central hypothalamic-pituitary-gonadal (HPG) axis activity over the lifetime. The first occurs during fetal life, the second—termed “mini-puberty”—in the first months after birth, and the third at puberty. After adolescence, the axis remains active all through adulthood. Congenital hypogonadotropic hypogonadism (CHH) is a rare genetic disorder characterized by a deficiency in hypothalamic gonadotropin-releasing hormone (GnRH) secretion or action. In cases of severe CHH, all 3 waves of GnRH pulsatility are absent. The absence of fetal HPG axis activation manifests in around 50% of male newborns with micropenis and/or undescended testes (cryptorchidism). In these boys, the lack of the mini-puberty phase accentuates testicular immaturity. This is characterized by a low number of Sertoli cells, which are important for future reproductive capacity. Thus, absent mini-puberty will have detrimental effects on later fertility in these males. The diagnosis of CHH is often missed in infants, and even if recognized, there is no consensus on optimal therapeutic management. Here we review physiological mini-puberty and consequences of central HPG axis disorders; provide a diagnostic approach to allow for early identification of these conditions; and review current treatment options for replacement of mini-puberty in male infants with CHH. There is evidence from small case series that replacement with gonadotropins to mimic “mini-puberty” in males could have beneficial outcomes not only regarding testis descent, but also normalization of testis and penile sizes. Moreover, such therapeutic replacement regimens in disordered mini-puberty could address both reproductive and nonreproductive implications.

## Graphical Abstract

**Graphical Abstract:**
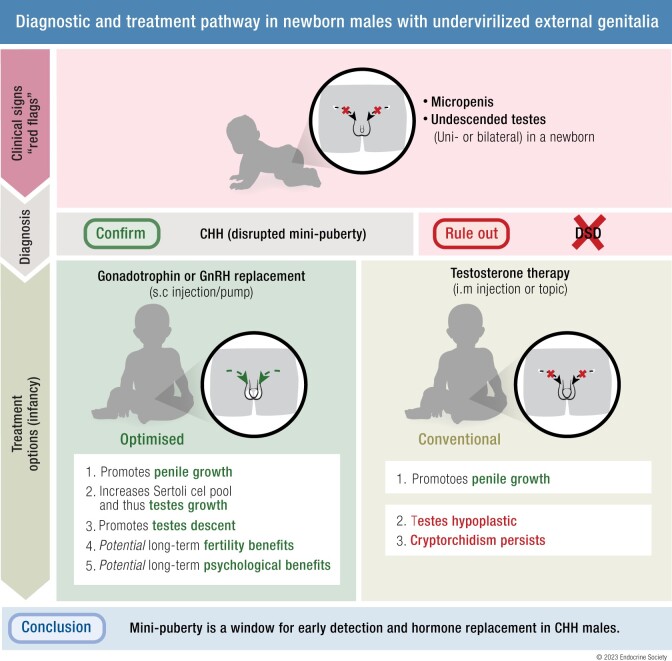

**ESSENTIAL POINTS**

- Mini-puberty refers to the second physiological wave of activation of the hypothalamic-pituitary-gonadal axis (the first being activation that occurs in utero, and the third adolescent puberty)
- Mini-puberty is a particularly important period in males, where growth and development of the testes occurs with consequent increased capacity for sperm production in adult life
- In cases of congenital hypogonadotropic hypogonadism (CHH), characterized by a deficiency in secretion of gonadotropin-releasing hormone, mini-puberty is disrupted, resulting in testicular immaturity
- Mini-puberty is a key window of opportunity for diagnosis of male CHH, as signs of micropenis and/or cryptorchidism can alert the clinician to an underlying disorder.
- If untreated, patients with CHH often present with delayed or absent adolescent puberty, and subsequent substantial consequences including infertility, decreased bone mineral density, negative self-image, and decreased mental health
- Conventional treatment of male infants with CHH is with testosterone therapy, which promotes penile growth, but physiological replacement of minipuberty with gonadotropins has additional advantages in promoting testicular descent, increasing the Sertoli cell pool, and may have long-term benefits for fertility
- There is potential to improve outcomes for males with CHH through earlier diagnosis, identification of red flags, and participating in further research into optimal therapeutics, ideally randomized trials or as part of international registries

## Definition of Mini-Puberty

The term “*mini-puberty*” refers to the transient activity of the hypothalamic-pituitary-gonadal (HPG) axis that occurs after birth. In males, this activity is most prominent at 2 to 3 months postnatal age and ends at around 6 months. In females, the axis may be active until age 2 to 4 years (1-3). During mini-puberty, concentrations of gonadotropin hormones rise secondary to pulsatile release of hypothalamic gonadotropin-releasing hormone (GnRH). This leads to detectable serum concentrations of sex steroids, comparable to those seen in healthy adults.

## What Is the Significance of Mini-Puberty?

There is robust evidence that progression through mini-puberty is vital for the promotion of future reproductive capacity in males. In infant males this period is associated with growth and development of the testes and penis, as well as somatic changes. A particularly significant developmental event during mini-puberty in males is the change in the cellular composition of the testes, with an increase in the number of the immature Sertoli cells. Sertoli cells are part of the somatic compartment of the testes and are needed to support spermatogenesis and thus the production of mature spermatozoa at puberty and during adulthood. Spermatogenesis is a 2-step process, taking place in the seminiferous tubules; it includes meiosis of diploid spermatogonial stem cells to haploid pachytene spermatocytes and development of round spermatids to elongated spermatids (spermiogenesis).

In females, the prognostic role of gonadal steroidogenesis during mini-puberty, and specifically the effect on target tissues, is less clear (4). In contrast to males, the oocyte pool in females is already complete at birth, although gonadotropin stimulation of the female fetus during late gestation and postnatally may well be important for maturation and atresia of ovarian follicles.

## Why Focus on Mini-Puberty?

The infantile mini-puberty provides a unique window of opportunity for diagnosis of central disorders of hypothalamo-pituitary gonadal function and for early therapeutic intervention.

However, there is little agreement about the practical management of infants with severe GnRH deficiency associated with absent mini-puberty and current strategies do not promote future reproductive capacity.

There is a clear unmet need for research into the physiological importance of mini-puberty and the potential for improved diagnosis and management, especially in male infants with severe GnRH deficiency associated with absent mini-puberty. The optimization of the diagnosis and management of infants with disordered mini-puberty has the potential for major effect both in terms of patient care and health care economics. Particular questions include the following:

- How to achieve timely identification of infants with absent mini-puberty,
- How to replace central hormones to best mimic the physiology of mini-puberty,
- Whether and to what extent this will normalize the phenotype, and
- Whether in the long-term, this will promote or increase fertility, and finally
- Whether this will improve the psychological well-being of affected individuals in childhood and adult life.

This review is aimed at addressing these questions. Included are a description of the physiological development of the reproductive tract, from fetal life to adulthood, as well as the pathophysiology underlying conditions of GnRH deficiency, including congenital hypogonadotropic hypogonadism (CHH).

The review will further cover the associated hormonal changes that can be observed during mini-puberty in health and in conditions of congenital GnRH deficiency and discuss consequences for affected individuals in adolescence and in later life. Further, it aims to provide a possible approach to the clinical assessment of males suspected to be affected by GnRH deficiency at infancy and review currently available options for central hormone replacement during absent male mini-puberty with recommendations for further study.

## Physiological Development of the Reproductive Axis

### Role of the Hypothalamic-Pituitary-Gonadal Axis

Gonadal function is regulated by hormones of the central HPG axis. GnRH is secreted in a pulsatile fashion into the hypophyseal portal venous blood by the “GnRH pulse generator,” which consists of specialized neurons in the human hypothalamus (5). GnRH pulses exert a stimulating effect on GnRH receptors that are located on gonadotropic cells of the anterior pituitary gland. In response, the gonadotropins luteinizing hormone (LH) and follicle-stimulating hormone (FSH) are cosecreted into the bloodstream. The intensity of pituitary secretion of both pituitary hormones is differentially modulated by the amplitude and frequency of GnRH pulses. Male and female gonads respond to gonadotropin-stimulation by secreting sex steroid hormones.

In females, ovarian theca cells produce androstenedione and testosterone. These hormones diffuse to the neighboring granulosa cells, where they are used as precursors for estrogen synthesis (6). FSH stimulates estrogen secretion by granulosa cells, which in turn induces a peak of pituitary LH secretion that induces ovulation. Thereafter, luteinized granulosa and theca cells produce progesterone and estrogens on stimulation by both gonadotropins.

In males, testicular Leydig cells produce and secrete testosterone on LH stimulation. Testosterone, together with its 5-α reduced form, 5-dihydrotestosterone (DHT), are the key bioactive androgens (7). FSH stimulates testicular Sertoli cells with a 2-fold effect (8). Specifically, during mini-puberty and early puberty, Sertoli cells respond to FSH stimulation by undergoing mitotic divisions. During later puberty and adulthood, the role of Sertoli cells is to form the niche that nurtures germ cells on FSH stimulation, to enable spermatogenesis. Spermatogenesis includes meiosis of diploid spermatogonia to haploid pachytene spermatocytes and completion of spermatogenesis through development of elongated spermatids from round spermatids (spermiogenesis). This results in the production of mature spermatozoa (9).

Human HPG axis function is modulated by feedback mechanisms of the gonads to the hypothalamus and pituitary, including both positive and negative mechanisms. While testosterone, estradiol, progesterone, and inhibins act as inhibitors on GnRH, LH, and FSH secretion, activins exert a positive feedback (10).

In females, the feedback of estradiol on pituitary LH secretion is 2-fold: While stimulatory in the first phase of the menstrual cycle, to allow for the LH surge at ovulation, it is inhibitory in the second phase (11).

There are 3 physiologic waves of central HPG axis activity over the lifetime. The first occurs during fetal life, the second in the first months after birth (“mini-puberty”), and the third at puberty. From then on, the axis remains active all through adulthood.

### Antenatal Activation of the Hypothalamic-Pituitary-Gonadal Axis

During fetal development, GnRH neurons originating from the epithelium of the nasal placode migrate through the lamina cribrosa into the hypothalamus (12). This migration is accompanied by olfactory neurons and accomplished by 14 weeks’ gestation (weeks since the last menstrual period of the mother) (13). The exact timing of the initiation of pulsatile GnRH secretion is uncertain, but an in vitro model using fetal hypothalamic explants from 20 to 23 weeks’ gestation showed GnRH pulsatility (14).

The pituitary gland is derived from the neural ectoderm, which evolves into the posterior lobe, and the surface ectoderm, which develops into the Rathke pouch and the anterior and intermediate lobes (15). The α gonadotropin subunit and LH are both detectable from 9.5 weeks’ gestation in pituitary extracts (16). The β subunits of FSH and LH are detectable from around 12 weeks onward (17). From 15 to 25 weeks, gonadotropins are detectable in female pituitaries, while in males, the peak of HPG axis activity in gonadotropin-containing cells is observed at 26 weeks’ gestation. In males, LH activity predominates over FSH while for females LH and FSH concentrations are more similar (2).

In males, this HPG activation is important in facilitating testicular descent, a complex process depending on anatomical and endocrine components. The testes typically migrate from their initial site in the upper-dorsal abdomen (the urogenital ridge) to the scrotum by full term. From as early as 6 weeks’ gestation, Leydig cell precursors in the gonads begin to secrete testosterone (18). At first, prior to the 15th week of gestation, this occurs independently of LH in response to placental hCG (19). Later in gestation, Leydig cells respond to stimulation by LH on fetal GnRH activity (20). Secretion of testosterone peaks between 12 to 16 weeks’ gestation (18). Independence of early fetal testosterone secretion from central HPG axis activity is the reason why males with severe GnRH or pituitary gonadotropin deficiency in utero do not develop ambiguous genitalia.

The role of testosterone in the male fetus is 2-fold: (i) facilitating testicular descent, and (ii) inducing penile development. The evidence for this in humans is provided by the observation that androgen insensitivity induces incomplete testis descent (21, 22), while the evidence that penile and scrotal development from the primitive urethral folds is additionally controlled by the more bioactive metabolite DHT stems from the observation that males with 5-α-reductase deficiency have penile hypoplasia (and undescended testes; UDT) (23, 24).

Insulin-like factor 3 (INSL3), a peptide hormone produced by Leydig cells on LH stimulation, is also vital for testicular descent as it facilitates differentiation and growth of the genitoinguinal ligament (gubernaculum) (25). In mice the absence of INSL3 leads to cryptorchidism (26). Lower concentrations of INSL3 are seen in humans with congenital GnRH deficiency (27). FSH has a vital role in utero in facilitating Sertoli cell proliferation in the testes (28). It amplifies Sertoli cell production of inhibin B and antimüllerian hormone (AMH). AMH causes regression of the müllerian ducts from 8 weeks’ gestation onward (29).

In females, fetal ovaries do not express FSH receptors until later gestation (30) and normal ovarian development can occur until 34 weeks even in an anencephalic fetus, indicating independence of these developmental processes from central HPG axis stimulation. The development of an ovarian reserve is driven by estrogens produced by the placenta.

Primordial follicles develop in the fetal ovaries before gestational week 13 and reach a maximum at gestational week 34, then remain stable at least until age 8 months (31). Thereafter there is a gradual decline of the follicle pool until around age 50 years. While the female ovarian pool contains around 2 million follicles at birth, this pool declines to 0.5 million at age 5 years and 0.3 million at a postpubertal age (32). It then remains stable until the second part of the third decade of life, then progressively vanishes until menopause, with an acceleration of the process after age 38 years (33).

### Postnatal Mini-Puberty in Males

Mini-puberty is the second of the 3 key phases of activation of the HPG axis (3, 34). It is a period of increased gonadotropin and sex hormone secretion and occurs during the first 6 months of life in boys. This phase of HPG activation is thought to be important in males for postpubertal reproductive capacity, as gonadotropin stimulation of the testes acts to increase the number of Sertoli cells and germ cells, which are key determinators of future potential for spermatogenesis (25, 35-37). As a result, testicular volumes increase concurrent with mini-puberty.

Mini-puberty was first described in boys in the early 1970s (38). The inhibition of placental estrogen on fetal pituitary FSH and LH secretion ceases at parturition, and thus constitutes the starting point of mini-puberty. There is an immediate increase, within minutes of birth, in testosterone, with a peak at 2 to 3 hours, followed by a decline after 6 to 12 hours of life (39, 40). After this initial peak in testosterone, there is a decline in all HPG hormones in the male and the female for about 1 week, before increasing again (35, 41). This is followed by mini-puberty, lasting about 6 months in boys. LH increases during the second week of life and peaks during the second to tenth weeks (42, 43). It is followed by an increase in testosterone produced by the Leydig cells, reaching adult concentrations at age 2 to 3 months before it declines, and returns to low concentrations at about age 6 months (44). Leydig cell production of INSL3 on LH stimulation peaks at age 1 month; this further stimulates testicular descent or maintains final testicular position in the scrotum (25, 35). FSH increases during the second week, reaches its maximum at 10 to 15 days, and declines after 3 months (35, 42, 43). The rise in FSH is associated with an increase in AMH and inhibin B produced by the proliferating Sertoli cell pool. The serum concentration of both peptides peaks at around age 4 months and stays elevated into the childhood years (44-46). Reference ranges for key biochemical parameters during mini-puberty are described in Table 1.

**Table 1.** Reference ranges for key biochemical parameters during mini-puberty

Parameter	Age	Median/Mean	P2.5-p97.5	Sample size	Citation
**Males**					
LH, IU/L	2.0-3.5 mo	1.71 (geometric mean)	0.62-4.08	581	(2)
	3 mo	1.74 (median)	0.90-2.64 (range)	15	(44)
	3.5-5.0 mo	1.40 (geometric mean)	0.54-3.32	166	(2)
	6 mo	0.36 (median)	0.16-1.07 (range)	15	(44)
FSH, IU/L	0 mo	0.70 (median)	0.32-1.61 (range)	15	(44)
	2.0-3.5 mo	1.19 (geometric mean)	0.41-3.02	578	(2)
	3 mo	1.79 (median)	0.90-2.93 (range)	15	(44)
	3.5-5.0 mo	1.11 (geometric mean)	0.42-2.68	165	(2)
	6 mo	0.96 (median)	0.29-1.78 (range)	15	(44)
Testosterone (RIA), nmol/L	2.0-3.5 mo	3.04 (geometric mean)	0.69-7.60	592	(2)
	3 mo	4.02 (median)	1.83-6.54 (range)	15	(44)
	3.5-5.0 mo	1.97 (geometric mean)	<LOD-7.01	168	(2)
	6 mo	<0.23	—	15	(44)
Testosterone (LC-MS/MS), nmol/L	2.0-3.5 mo	4.75 (geometric mean)	1.35-11.3	251	(2)
	3.5-5.0 mo	2.25 (geometric mean)	0.32-9.65	175	(2)
Estradiol, pmol/L	2.0-3.5 mo	<LOD (geometric mean)	<LOD-47	571	(2)
	3.5-5.0 mo	<LOD (geometric mean)	<LOD-49	160	(2)
Inhibin B, pg/mL	0 mo	140 (median)	87-243 (range)	15	(44)
	2.0-3.5 mo	379 (geometric mean)	229-631	579	(2)
	3 mo	361 (median)	254-513 (range)	15	(44)
	3.5-5.0 mo	379 (geometric mean)	222-662	162	(2)
	6 mo	330 (median)	204-427 (range)	15	(44)
	0-12 mo	238 (median)	99-439	N/A	(47)
AMH, pmol/L	0-2 d	258 (median)	73-629	51	(48)
	3-7 d	486 (median)	119-1112	45	(48)
	8-10 d	563 (median)	193-1074	14	(48)
	11-20 d	522 (median)	211-988	37	(48)
	21-28 d	505 (median)	201-1055	26	(48)
	2.0-3.5 mo	1013 (geometric mean)	425-1810	48	(2)
	3.5-5.0 mo	1183 (geometric mean)	797-NA	12	(2)
	29-364 d	663 (median)	288-1242	66	(48)
**Females**					
LH, IU/L	2.0-3.5 mo	<LOD (geometric mean)	<LOD-0.98	432	(2)
	3 mo	0.08 (median)	<0.05-1.00 (range)	15	(44)
	3.5-5.0 mo	<LOD (geometric mean)	<LOD-1.25	110	(2)
	6 mo	<0.05 (median)	<0.05-0.29 (range)	15	(44)
FSH, IU/L	0 mo	0.06 (median)	<0.06-0.69 (range)	15	(44)
	2.0-3.5 mo	3.98 (geometric mean)	1.23-17.4	435	(2)
	3 mo	2.57 (median)	0.48-24.0 (range)	15	(44)
	3.5-5.0 mo	3.93 (geometric mean)	1.30-17.7	111	(2)
	6 mo	3.05 (median)	1.68-8.71 (range)	15	(44)
Testosterone (RIA), nM	2.0-3.5 mo	<LOD (geometric mean)	<LOD-0.40	74	(2)
	3.5-5.0 mo	<LOD (geometric mean)	<LOD-NA	14	(2)
Testosterone (LC-MS/MS), nM	2.0-3.5 mo	<LOD (geometric mean)	<LOD-0.21	165	(2)
	3.5-5.0 mo	<LOD (geometric mean)	<LOD-0.17	125	(2)
Estradiol, pmol/L	2.0-3.5 mo	29 (geometric mean)	<LOD-79	455	(2)
	3.5-5.0 mo	31 (geometric mean)	<LOD-98	113	(2)
Inhibin B, pg/mL	0 mo	<18 (median)	—	15	(44)
	2.0-3.5 mo	62 (geometric mean)	<LOD-184	423	(2)
	3 mo	32 (median)	<18-226 (range)	15	(44)
	3.5-5.0 mo	62 (geometric mean)	<LOD-174	106	(2)
	6 mo	80 (median)	<18-208 (range)	15	(44)
AMH, pmol/L	0-28 d	0.37 (median)	0.002-4.08	24	(48)
	2.0-3.5 mo	11 (geometric mean)	<LOD-49	339	(2)
	3.5-5.0 mo	15 (geometric mean)	2-46	67	(2)
	29-364 d	7.36 (median)	0.05-38.58	17	(48)

Abbreviations: AMH, antimüllerian hormone; FSH, follicle-stimulating hormone; LC-MS/MS, liquid chromatography with tandem mass spectrometry; LH, luteinizing hormone; LOD, limit of detection; NA, not available; RIA, radioimmunoassay.

Androgen synthesis in the testicular Leydig cells during mini-puberty seems to result mainly from the backdoor pathway, as studies have shown that in urine, metabolites in the Δ 4 lyase pathway of steroidogenesis predominates rather than the Δ 5 pathway (49). Interestingly, there are large variations in total testosterone concentrations during mini-puberty (35, 50).

The postnatal increase in testosterone seems to be important for further development of the external male genitalia, specifically penile growth (51). The increase in FSH is important for seminiferous tubule development, with mitotic proliferation of immature Sertoli cells and germ cells (36, 52, 53). The increase in cell numbers in the testes is seen as a moderate increase in testicular volume, which peaks at age 4 to 5 months, increasing in volume by around 40% relative to size at birth, before receding by age 1 year, resulting after mini-puberty is completed to slightly above the volume at birth (35). Normal values for testicular volume and penile length at birth and in the first months of life are described in Table 2. The Sertoli cells not only expand in numbers but also support gonocyte maturation and development into spermatogonial stem cells. Importantly, Sertoli cells start to express the androgen receptor only from about age 1 year; there is a progressive increase in its expression that reaches its maximum at about 4 to 5 years (54). Thus, while during mini-puberty there is an intratesticular testosterone surge produced by the Leydig cells, there is no corresponding meiotic response from the Sertoli cells and thus spermatogenesis does not occur in infancy (53).

**Table 2.** Normal penile length and testicular volumes during the mini-puberty period

Citation	Average	Population	Sample size	Age	Ethnicity/Country of study
**Penile length**					
Schonfeld et al 1942 (55)	Median 3.75 cm	Healthy	125	0-5 m	White
Feldman et al 1975 (56)	Mean 3.5 cm	Healthy	37	Newborn, term	United States
Suttan-Assin et al 1989 (57)	Mean 2.86 ± 0.23 cm	Healthy	336	Newborn, term	Indonesia
Cheng and Chanoine 2001 (58)	Mean 3.4 ± 0.3 cm	Healthy	40	Newborn, term	White
Cheng and Chanoine 2001 (58)	Mean 3.1 ± 0.3 cm	Healthy	40	Newborn, term	Chinese
Cheng and Chanoine 2001 (58)	Mean 3.6 ± 0.4 cm	Healthy	25	Newborn, term	East Indian
Al-Herbish 2002 (59)	Mean 3.55 ± 0.57 cm	Healthy	379	Newborn, term	Saudi Arabia
Boas et al 2006 (51)	Mean 3.49 ± 0.40 cm	Healthy	1962	Newborn, term	Danish and Finnish
Tomova et al 2010 (60)	Mean 3.55 ± 0.46 cm	Random population sample	310	0-11 mo	Bulgaria
Matsuo et al 2014 (61)	Mean 3.06 ± 0.26 cm	Healthy	547	Newborn, term	Japanese
Singal et al 2016 (62)	Mean 3.31 ± 0.38 cm	Healthy	553	Newborn, term	India
El-Ammawi et al 2018 (63)	Mean 3.5 ± 0.6 cm	Clinic attendees (unrelated)	37	Newborn	Egypt
El-Ammawi et al 2018 (63)	Mean 3.7 ± 0.6 cm	Clinic attendees (unrelated)	84	1-4 mo	Egypt
Van der Straaten et al 2020 (64)	Mean 3.12 ± 0.54 cm	Healthy	174	0-1 mo, term	Europe (various)
Van der Straaten et al 2020 (64)	Mean 3.11 ± 0.52 cm	Healthy	96	1-6 mo	Europe (various)
**Testicular volume**					
Schonfeld et al 1942 (55)	Median 0.52 cc	Healthy	125	0-5 mo	White, United States
Suttan-Assin et al 1989 (57)	Mean 1.25 ± 0.43 mL (orchidometer)	Healthy	336	Newborn, term	Indonesia
Kuijper et al 2008 (65)	Mean 0.44 ± 0.03 cm^3^ (ultrasound)	Healthy	Not stated (subgroup)	5 mo	Mixed (White, African, Asian, Mediterranean)
Tomova et al 2010 (60)	Mean 0.99 ± 0.19 mL (right); mean 1.01 ± 0.11 mL (left) (orchidometer)	Random population sample	310	0-11 mo	Bulgaria
Chikani et al 2016 (66)	Mean 1.74 ± 0.62 mL (orchidometer)	Healthy	811	Newborn, term	Nigeria
Lawal et al 2016 (67)	Mean 1.28 ± 1.03 mL (ultrasound)	Healthy	138	0-12 mo	Nigeria
Liu et al 2021 (68)	Mean 0.43 ± 0.13 mL (left) mean 0.44 ± 0.14 mL (right) (ultrasound)	Healthy	93	0-11 mo	China
Saneja et al 2021 (69)	Mean 0.86 ± 0.16 mL (orchidometer)	Healthy	409	Newborn, term	India

### Postnatal Mini-Puberty in Females

The relevance of elevated reproductive hormones for male genital development and future fertility during mini-puberty is well described, but its role for fertility is less clear in female individuals, as the primordial follicular pool is completed antenatally and stable along the major period of female mini-puberty until at least age 8 months (31).

Elevated serum estrogen concentrations occurring during mini-puberty are associated with early mammary and uterine growth. Female mini-puberty thus may induce physiologic breast development, which usually resolves by age 2 to 3 years but may persist into mid-childhood (premature thelarche). These postnatal changes in the estrogenic target tissues in response to HPG axis activation and secretion of endogenous ovarian steroid hormones reflect ovarian follicular function and development (70, 71).

Information from murine studies in female models hints that there may be a physiological effect of mini-puberty on structures related to reproductive success. These studies demonstrate that gonadotropins are essential for early follicular development in mice and suggest further possible roles such as establishment of coordinated GnRH pulsatility, sexual and maternal behavior, alongside uterus and mammary gland development (72). However, since maturation of germ cells does not seem to undergo a critical maturational process affecting future female fertility during mini-puberty, the later sections of this review focus primarily on male aspects of mini-puberty.

### Postnatal Hypothalamic-Pituitary-Gonadal Activation in Premature and Small-for-Gestational-Age Infants

In preterm infants, mini-puberty is slightly different compared to full-term infants. In premature infants born between 28 and 42 weeks’ gestational age, the postnatal surge in LH and FSH is higher and more prolonged both in males and females (73).

In preterm boys, LH and testosterone, as well as FSH, reach higher concentrations compared to full-term male infants at 1 to 3 months (74, 75). In addition, preterm boys may have significantly faster testicular and penile growth. Preterm girls also show an exaggerated HPG axis activity, with a more pronounced increase in FSH, which is also more prolonged compared to full-term girls. These high concentrations of LH and FSH peak at around 32 weeks’ corrected age (75, 76). The FSH surge results in transient ovarian stimulation with increased number of ovarian follicles at the antral phase and higher concentrations of AMH (71, 77). Similarly exaggerated mini-puberty profiles have been observed in infants born small for gestational age (78, 79).

In cases of extreme preterm birth, the normal antenatal activation of the HPG axis can be highly disrupted. In preterm girls, between 25 to 30 weeks’ gestation, the excess of estrogen and gonadotropins has been linked to cases of preterm ovarian hyperstimulation syndrome, with features including breast enlargement, swollen external genitalia including clitoris, labia majora and minora, and vaginal bleeding, in association with elevated estradiol, LH, and FSH and large follicular ovarian cysts (80-83). However, the phenotype and biochemical parameters self-resolve over time without intervention (84) and information on consequences in later life is unknown or inconsistent. Several studies have found no relationship between being born prematurely or small for gestational age and gonadal function (85-87).

### Hypothalamic-Pituitary-Gonadal Axis Quiescence After Mini-Puberty, Adolescent Puberty, and Beyond

After the completion of mini-puberty in children of both sexes there is a period of dormancy of the HPG axis until the start of adolescent puberty (88-91). During this period, GnRH secretion is minimal, with undetectable concentrations of LH and sex steroids except with the use of ultrasensitive assays.

Puberty is the third phase of HPG axis activity and takes place during adolescence. From then on, the axis remains active throughout life. During puberty, boys and girls mature physically and psychologically, and attain reproductive capacity by its completion (92-94).

In boys, development of male secondary sexual characteristics (virilization) begins between ages 9 and 14 years. Its beginning is defined and thus recognizable by the increase in testicular volumes to 4 mL or above. At initiation of puberty, this testicular growth results from an increase in Sertoli cell volumes and peritubular myoid cells. Thereafter, the increase in size is exclusively driven by the elongation and the thickening of seminiferous tubules, both resulting from activation of spermatogenesis.

In girls, puberty begins between ages 8 and 13 years, with the first external sign being thelarche (breast development Tanner B2), triggered by increases in estrogen leading to expansion of the lactiferous ducts into the mammary fat pad. Estrogens also induce uterine growth. Progesterone secreted after ovulation leads to alveolar bud development (95).

Of note, both in males and females, development of pubic hair and sebaceous and apocrine glands initially results from adrenal androgen secretion within the zona reticularis during adrenarche, a process that is gonadotropin independent and that starts from ages 6 to 8 years onward. Adrenarche is indexed by a rise in the blood level of dehydroepiandrosterone sulfate (DHEAS). Recently, 11-ketotestosterone has been recognized to significantly contribute to androgenic bioactivity (96). However, from mid-puberty onward, pubic hair growth is enhanced and maintained also by androgens of gonadal (testicular/ovarian) origin.

The aforementioned physiognomic changes induced by adolescent puberty are the result of complex regulatory processes within the neuroendocrine network of the hypothalamus. With the onset of puberty, there is a sustained increase in frequency and amplitude of the pulsatile release of GnRH. This is secondary to a change in the balance of inhibitory and excitatory inputs to GnRH neurons from Kisspeptin/ NeurokininB/ Dynorphin (KNDy) neurons and additional upstream regulatory elements. As excitatory inputs become dominant, GnRH is increasingly secreted in a pulsatile fashion into the hypophyseal portal blood (97, 98). As a result, LH and FSH from the gonadotropes in the anterior pituitary are released into the peripheral bloodstream.

In males, while LH stimulates the Leydig cells in the testes to synthesize and secrete testosterone in a similar manner as during mini-puberty, FSH, in concert with intratesticular testosterone acting via the now-expressed androgen receptors, stimulates Sertoli cells to promote spermatogenesis, whereby one Sertoli cell supports the maturation of 18 to 20 germ cells at different spermatogenic stages (99, 100). Sertoli cells regulate the passage of growth factors and nutrients across the seminiferous epithelium to the germ cells, produce signaling molecules to regulate spermatogenesis, and organize mature spermatid passage to the tubular lumen (101, 102).

**Figure 1.**
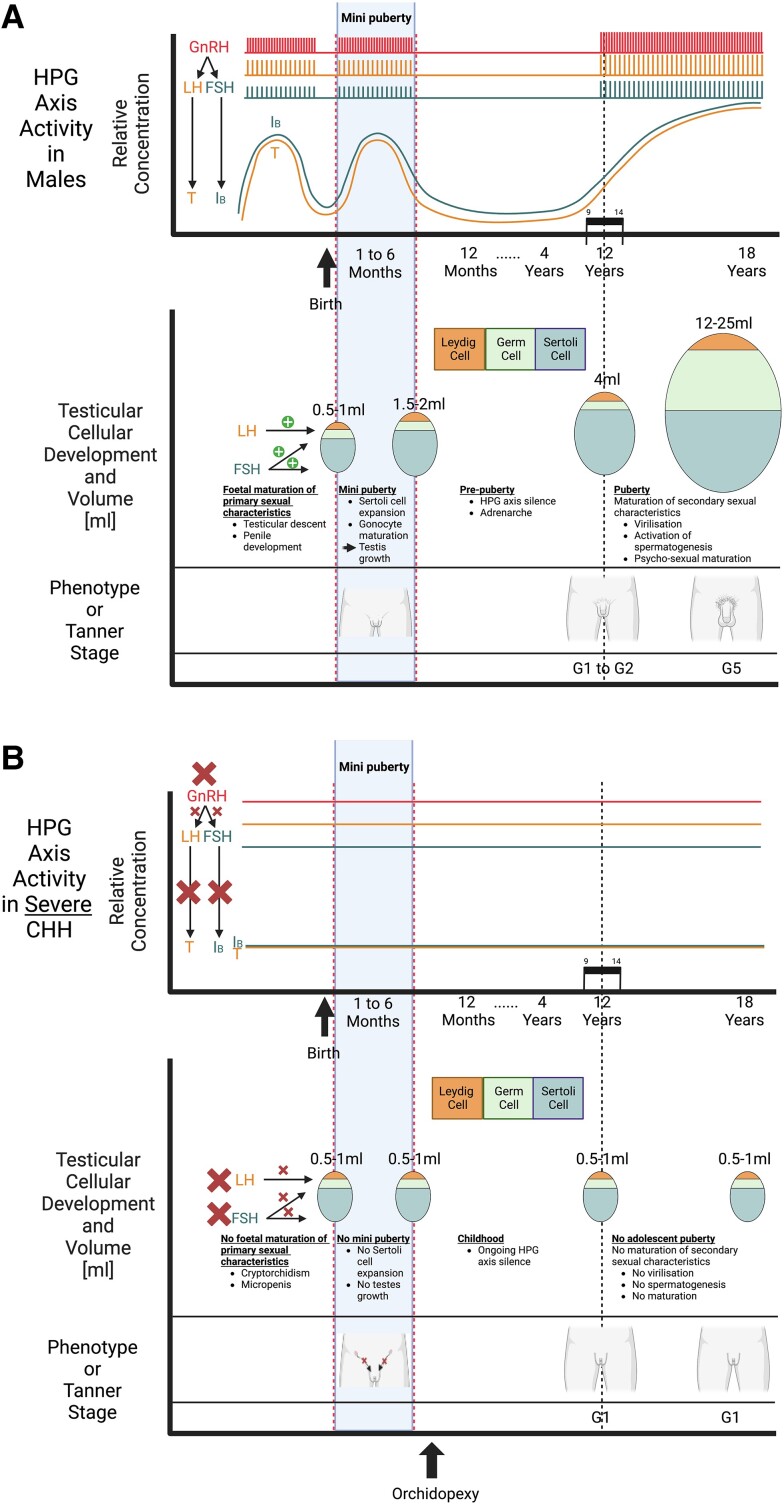
A, Physiologic waves of male hypothalamic-pituitary-gonadal (HPG) axis activity, with consequences for testicular growth and cellular composition of the testes. Created with BioRender. B, Absent HPG axis activity in males with severe congenital hypogonadotropic hypogonadism, with consequences of reduced testicular growth and development. Created with BioRender.

**Figure 2.**
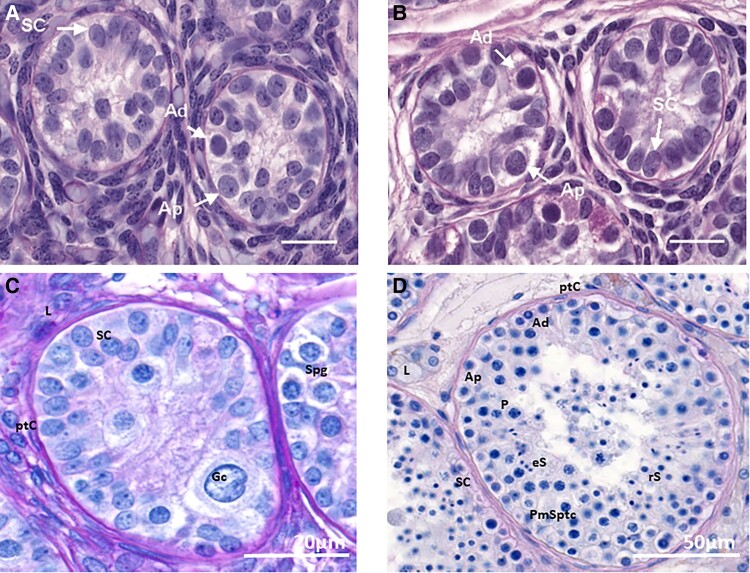
Histology of A to C, primate testes and D, human testes. A, Photomicrograph of testis of 1- to 2-day-old neonatal rhesus monkey showing Sertoli cells (SC) and type A pale and type A dark spermatogonia (Ap and Ad); B, Photomicrograph of testis of 4- to 5-month-old infant rhesus monkey, post mini-puberty, showing increased numbers of Sertoli cells. C, Sections of seminiferous tubules in a prepubertal healthy marmoset, histologically identical to that of a prepubertal human testis containing spermatogonia and Sertoli cells. The spermatogonia have not yet undergone any meiotic divisions. D, Sections of seminiferous tubules of a testis in a healthy postpubertal adolescent. The tubules contain all stages of spermatogonial meiosis, including elongated spermatids (mature sperm). Ad, A dark spermatogonia; Ap, A pale spermatogonia; eS, elongated spermatid; Gc, gonocyte; L, Leydig cell; P, pachytene spermatocyte; PmSptc, premeiotic spermatocyte; ptC, peritubular myoid cell; ; rS, round spermatid; SC, Sertoli cell; Spg, spermatogonia. Both (A) and (B) from Simorangkir et al (36), (C) and (D) authors’ own images. Scale bar A, B + C is 20 µm, D is 50 µm.

During the process of testicular maturation at puberty, the gonads increase further in size (Fig. 1A and Fig. 2). This testicular growth results from elongation and thickening of the seminiferous tubules, which in turn results from the activation of spermatogenesis. Final sizes of the testes are determined by germ cell numbers, which in turn depend on the number of Sertoli cells—finally established at the beginning of puberty—that nurture the gonadal stem cells (103, 104). Full activation of spermatogenesis is accomplished at the end of pubertal maturation. Mature testes of fertile men produce semen that contains more than 15 million elongated spermatozoa per milliliter (105, 106). From adolescence onward and throughout adulthood, the serum concentration of inhibin B (secreted by Sertoli cells) is known to correlate with spermatogenesis (107, 108).

In females, GnRH pulsatility triggers LH and FSH release, which act synergistically to stimulate follicular growth; ovulation is induced by an LH peak. The ovulated follicle then differentiates into the corpus luteum, which synthesizes progesterone (109, 110). Thecal cells in the ovary produce androstenedione and testosterone; these androgens are then converted into estradiol in granulosa cells (111). Menarche occurs around 2 to 3 years after thelarche (112), once the biphasic menstrual cycle has first been established.

Both in males and females, sex steroids act in concert with growth hormone on the bone to induce a pubertal growth spurt (113). This is terminated by estrogens inducing the fusion of the growth plates (114). In addition, sex hormones are essential for bone mineralization, and thus acquisition of peak bone mass by age 20 to 24 years (115, 116).

## Conditions With Disordered Mini-Puberty

### Genetic Variation Associated With Absent or Disordered Mini-Puberty

Pathogenic variants in more than 50 genes have been described as causal of or contributory to the phenotype of congenital GnRH deficiency and associated syndromes linked to absent or disordered mini-puberty (117). Variants of interest in relevant genes are found in up to 50% of research cohorts with CHH (118-122), even among adult patients with no other distinctive phenotypic features (121, 123).

However, genetic testing in infant cohorts with CHH has not been well described. Many centers use either panel (targeted next-generation sequencing) or whole-exome sequencing testing of a variable number of these genes for the genetic diagnosis of CHH (124). Custom or virtual panels reporting large numbers of genes are more likely to report an identified pathogenic variant in up to 60% of cases, whereas positive results fall below 15% in centers using small panels.

The UK National Health Service (England) Genomic Medicine Service has a gene panel for hypogonadotropic hypogonadism (panel R148 v3.2, last updated March 22, 2023), with 22 genes included based on high levels of evidence that if identified will be reported to the patient's clinician, as well as 23 additional genes that are tested for research purposes (117). The 22 genes with high levels of evidence and/or strong biological plausibility are described in Table 3 alongside descriptions of function and frequency. The majority of genetic inheritance is biallelic (recessive) autosomal, but X-linked biallelic and monoallelic autosomal (dominant) inheritance patterns are also seen. While initial discoveries described CHH as a monogenic disease, increasingly patients are described with multiple deleterious variants in a digenic or oligogenic inheritance pattern (121, 125).

**Table 3.** Key genes described in congenital hypogonadotropic hypogonadism (Kallmann syndrome or normosmic)—based on Genomics England Hypogonadotropic Hypogonadism Panel (3.2)

Gene	Role	Estimated frequency in CHH	KS or nCHH	Location	Inheritance	Citation
*ANOS1 (KAL1)*	Encodes protein anosmin-1, vital for olfactory and GnRH neuronal migration	7.1%	KS	Xp22.31	X-linked recessive	(126)
*CHD7*	Encodes protein chromodomain helicase DNA binding protein 7, associated with CHARGE syndrome	16%	Both (also CPHD)	8q12.1	Autosomal dominant	(127)
*FGF8*	Encodes FGF8, signaling factor involved in GnRH neuron specification and development of olfactory system	1.3%	Both (also CPHD)	10q24.32	Autosomal dominant or recessive	(128)
*FGFR1*	Encodes receptor involved in signaling for GnRH neuron specification and development of olfactory system	9.0%	Both (also CPHD)	8p11.23	Autosomal recessive	(128)
*FSHB*	Encodes FSH subunit β protein	<1%	nCHH	11p14.1	Autosomal recessive	(129)
*GNRH1*	Encodes GnRH protein	1.7%	nCHH	8p21.2	Autosomal recessive	(130)
*GNRHR*	Encodes GnRH receptor	4.7%	nCHH	4q13.2	Autosomal recessive	(131)
*IL17RD*	Encodes IL17RD protein, which interacts with FGF8	2.1%	KS	3p14.3	Autosomal recessive	(125)
*KISS1R*	Encodes kisspeptin receptor, with kisspeptin being the main regulator of GnRH neuron activation and secretion	2.0%	nCHH	19p13.3	Autosomal recessive	(131)
*KLB*	Encodes β-Klotho, obligate coreceptor for FGF21	4%	Both	4p14	Autosomal dominant	(132)
*LEP*	Encodes hormone leptin, which regulates neuroendocrine function and reproduction	<1%	nCHH	7q32.1	Autosomal recessive	(121)
*LEPR*	Encodes receptor for leptin, thereby being involved in regulation of neuroendocrine function and reproduction	<1%	nCHH	1p31.3	Autosomal recessive	(121)
*LHB*	Encodes LH subunit β protein	<1%	nCHH	19q13.33	Autosomal recessive	(133)
*NDNF*	Encodes neuron-derived neurotrophic factor, involved in neuron survival and migration	1.7%	KS	4q27	Autosomal dominant	(134)
*PLXNA3*	Encodes plexin receptor, Plexin-A3, which is involved in plexin signal transduction and neuronal axon guidance	2.3%*	Both	Xq28	X-linked recessive	(136)
*PROK2*	Encodes prokineticin 2, involved in migration and regulation of GnRH neurons and formation of olfactory bulb	2.1%	Both	3p13	Autosomal recessive	(137)
*PROKR2*	Encodes prokineticin 2 receptor, involved in migration and regulation of GnRH neurons and formation of olfactory bulb	5.2%	Both (also CPHD with PSIS)	20p12.3	Autosomal recessive	(137)
*SEMA3F*	Encodes the protein semaphoring-3F, which is involved in olfactory map topography	4.6%	Both	3p21.31	Autosomal dominant	(136)
*SOX10*	Encodes sex-determining region Y-box 10 protein, a regulator of neural crest development	1.5%	nCHH	22q13.1	Autosomal dominant	(138)
*TAC3*	Encodes neurokinin B, a peptide involved in regulation of GNRH release	1.0%	nCHH	12q13.3	Autosomal recessive	(131)
*TACR3*	Encodes receptor for neurokinin B, a peptide involved in regulation of GNRH release	2.6%	nCHH	4q24	Autosomal recessive	(131)
*WDR11*	Encodes WDR11, which is involved in hedgehog pathway gene expression and GnRH production	0.9%	Both	10q26.12	Autosomal dominant	(121)

*also found in high prevalence in healthy cohorts (135).

Abbreviations: CPHD, combined pituitary hormone deficiencies; FSH, follicle-stimulating hormone; GnRH, gonadotropin-releasing hormone; KS, Kallmann syndrome; LH, luteinizing hormone; nCHH, normosmic congenital hypogonadotropic hypogonadism; PSIS, pituitary stalk interruption syndrome.

Key genes are involved in the regulation of GnRH neuronal development, migration, and activity or in the regulation of GnRH homeostasis, secretion, or action on pituitary gonadotropic cells. While the frequency of burden of mutations in a particular gene vary from cohort to cohort, the most commonly identified loss-of-function variants are found in the *FGFR1*, *CHD7*, *PROKR2*, and *ANOS1* genes and in the gene encoding the GnRH receptor, *GNRHR* (123, 139).

Functional deficiency of genes important for fetal development and migration of GnRH neurons commonly underlies anosmic CHH (Kallmann syndrome) secondary to disruption of both olfactory and HPG axis neuronal migration. This is exemplified by the first gene to be identified in a human patient, *ANOS1* (known previously as *KAL1*), mutations in which are inherited in an X-linked fashion. Males with *ANOS1* loss-of-function mutations or deletions have a severe Kallmann syndrome phenotype with absent mini-puberty and a high prevalence of micropenis and cryptorchidism (119, 140). The severity of the phenotype is also mirrored by the response to gonadotropin treatment for fertilization in adolescence or young adulthood, with lowest spermatogenic responses in males with pathogenic *ANOS1* variants or *CHD7* variants and best responses in males with *GNRHR* mutations (139).

In contrast, females are affected by anosmic CHH only if both X chromosomes bear pathogenic variants in *ANOS1* or deletions including this gene.

Genes related to GnRH secretion, its upstream regulation, or downstream action on pituitary gonadotropes are usually contributory to CHH without additional anosmia. The peptide kisspeptin is established as a key regulator of GnRH activity, through activation of the kisspeptin receptor (encoded by *KISS1R*), which promotes pulsatile release of GnRH (141). Inactivating mutations in the *KISS1R* gene have been associated with CHH with disordered mini-puberty (142-145), and activating mutations with precocious puberty (146). The genes *TAC3* and *TACR3* encode neurokinin B (a peptide involved in regulation of GnRH release) and its receptor, and mutations in these genes are also described in patients with CHH (147). Mutations in *GNRHR* have been described in patients with CHH and a variable phenotype depending on the degree of loss of function of the receptor (148).

Downstream, genetic variants in the gene encoding the FSH receptor, *FSHR*, or affecting FSHβ subunit action were associated with significant changes in FSH, AMH, estradiol, and breast tissue size in a study of 402 infant females (149).

### Congenital Hypogonadotropic Hypogonadism in Males

CHH is a rare genetic condition whereby deficient production of GnRH from the hypothalamus leads to inadequate secretion of the gonadotropins LH and FSH from the anterior pituitary gland (Fig. 1B). Its pathogenesis and phenotype are best understood with recognition of the normal regulatory processes governing HPG activation, described earlier. The incidence is not well described, but CHH is more commonly reported in males than in females (150), and in males, it affects between 1 in 4400 and 30 000 live births (151, 152). When associated with anosmia or hyposmia this presentation is termed *Kallmann syndrome*, and is due to a shared defective migration of both the developing olfactory and GnRH neurons (153). CHH may occur in isolation, as Kallmann syndrome, or as part of a developmental disorder involving other pituitary hormone deficiencies (combined pituitary hormone deficiency, CPHD) (154).

In boys with severe CHH, lack of hypothalamic-pituitary stimulation of the testes during gestation and infancy leads to insufficient intrauterine maturation of the gonads and to defective testicular descent—from a position near the kidneys (the urogenital ridge) through the groin—into the scrotum. It also leads to undervirilization of the external genitalia, specifically hypoplasia of the penis (micropenis).

During puberty, there is absent or inadequate production of gonadal sex hormones and absent activation of spermatogenesis. This manifests as absent puberty. Thus, adolescence is the time point when CHH is most often diagnosed. This is particularly relevant for partial forms, where the external male genitalia may be normal at birth, mini-puberty may or not be abolished, and there may be spontaneous onset of puberty with subsequent pubertal arrest and therefore incomplete acquisition of pubertal milestones.

Clinical signs of inadequate GnRH secretion and hypogonadotropic hypogonadism in adolescence include persistence of testes of prepubertal (<3-4 mL) or early pubertal size, absence or partial virilization, lack or deficiency of pubertal muscle mass accrual, absence of facial hair growth, voice breaking, and decreased awakening of libido or sexual activity. Aside from low testes volumes, all of these symptoms are the consequence of persistently low testosterone concentrations (155).

Untreated, boys with pubertal failure may develop psychological symptoms including depression, anxiety, isolation, and body image concerns (156, 157).

Although CHH is for the most part recognized at adolescence or in young adult life (158), there is a distinct cohort of male patients with CHH, including those with Kallmann syndrome, who have symptoms of central HPG axis disturbance already present at birth. These are those with a more severe phenotype, in which all 3 waves of GnRH pulsatility are absent. These males could be identified at birth due to underdeveloped external genitalia. Micropenis and/or cryptorchidism of the male newborn are key “red flags” pointing to underlying severe CHH.

In males with CHH, the prevalence of UDT at birth is around 30%, while in those with Kallmann syndrome, the rate is around 70%. In individuals with *ANOS1* mutations or *CHD7* mutations, it is 90% to 100% (139). In rare cases, familial inheritance is identified in infancy. Although anosmia cannot be detected in infancy, brain imaging at this stage may identify aplastic or hypoplastic olfactory bulbs. Other associated features of CHH, not universally present, may also be identified in infancy, including ear, dental, and skeletal abnormalities, or renal agenesis.

Patients with Kallmann syndrome or severe CHH have been shown to respond more poorly to gonadotropin or GnRH therapy in adolescence than those with less severe GnRH deficiency in utero, with smaller posttreatment testicular volumes (159) and lower total sperm counts by the end of a treatment cycle (139, 160-164).

Cryptorchidism has been described as being a significant negative predictor for response to long-term GnRH therapy (165). In a study of 60 males with hypogonadotropic hypogonadism, aged 14 to 22 years, patients with congenital rather than acquired hypogonadotropic hypogonadism, and those with cryptorchidism, responded less well to treatment with hCG and recombinant FSH (rFSH), having lower final sperm counts (166). It has also been observed that congenital forms of hypogonadotropic hypogonadism require more time for gonadal stimulation with gonadotropins to induce spermatogenesis than forms of the disease acquired later in life (167).

These observations in male patients with CHH with the presence of phenotypic features in infancy raise the question of how to promote early identification and timely treatment of these patients by hormone replacement during mini-puberty. At present, “red flag” features of CHH are rarely attributed to central HPG axis failure and UDT and penile hypoplasia are thus often treated symptomatically (168). Attempts are made to increase penile size either with DHT gel preparations applied on the phallus or with intramuscular testosterone enanthate injected over a few months, while cryptorchidism is generally treated surgically.

To what extent lack of progression through mini-puberty, and its potential purpose in priming the testes, underlies poor response to treatment with GnRH or gonadotropins in adolescence or adulthood in males with CHH is not known at present. Therefore, there has been increasing interest in the potential of gonadotropins for the therapeutic mimicry of “mini-puberty.” It is hoped that there could be a resultant increase of future spermatogenic capacity of the testes.

The evidence regarding therapeutics for male CHH patients to replicate mini-puberty is scarce but shows potential for immediate positive outcomes. This will be discussed later.

## Congenital Hypogonadotropic Hypogonadism in Females

The genetic basis of CHH in females is similar to that of males, but only a minority of cases have X-linked inheritance (169). Therefore, the reported incidence in females is much lower. A nationwide study in Finland reported an estimated incidence of CHH in females at 1 in 125 000, compared to 1 in 30 000 males (152). It is important to emphasize that there is no mendelian genetic explanation for such a high sex ratio. In contrast, the ratio of affected males/females in kindreds having more than 1 affected individual seems to be lower than 4:1, with an equal number of affected males and females in some large pedigrees (170).

There are several factors that are likely to contribute to this difference in incidence, including the considerable underreporting of CHH in females, and prescription of the combined oral contraceptive pill in case of absent or arrested female puberty without previous hormonal or genetic evaluation. Female patients are significantly less likely to present with the associated “red flag” features of CHH such as anosmia, renal abnormalities, deafness, and bimanual synkinesis compared to males (169). This may in part be attributed to the X-linked inheritance of *ANOS1* mutations, which are often accompanied by renal agenesis. Females are more likely to present in adolescence, where the most common presenting complaint is primary amenorrhea in around 90% (158). As pubertal delay in females may be treated with sex steroids without discovery of the underlying etiology, this may also delay the diagnosis of hypogonadotropic hypogonadism, or prevent referral to tertiary centers, with patients instead receiving empirical treatment with combined oral contraceptives in primary care or with gynecology specialists.

Therefore, identification of females with CHH in the neonatal period remains challenging. This is echoed by the European Consensus Statement on CHH, which stated that there are no specific clinical signs of CHH in female neonates (120). Moreover, the significance of the deficient mini-puberty period for females with CHH (see Postnatal mini-puberty in females) is uncertain. However, the role for neonatal diagnosis to set patients onto a pathway to receive pubertal induction at the median age of normal pubertal onset, rather than late because of delayed diagnosis, is vitally important. When treatment is commenced in adolescence through use of estradiol, followed by combined estradiol and progesterone, this aims to induce breast development and other female secondary sexual characteristics, promote uterine maturation, and induce menses (158).

Fertility is induced in women with CHH with low doses of gonadotropins or short-term application of pulsatile GnRH for a few cycles. The success rates regarding conception after 6 cycles with gonadotropins are around 70%, and after the use of GnRH regimens are greater than 95% (171). Thus, absent puberty does not seem to have a detrimental effect on fertility of adult CHH female patients. The ovarian reserve in females with CHH is complete at birth, and postnatal differences can generally be successfully reversed by treatment in adult life (172). Consequently, at this point there has been minimal research into replacement of mini-puberty in females with CHH.

### Combined Pituitary Hormone Deficiencies

There are a large number of causes of neonatal CPHDs, which may be classified as developmental, syndromic, genetic, or secondary to direct damage during the perinatal or birth period (173).

The key genes associated with CPHD are described in Table 4. These defects lead to variable deficits in the release of LH, FSH, growth hormone, adrenocorticotropin (ACTH), thyroid Stimulating Hormone (or thyrotropin) (TSH), and prolactin from the anterior pituitary, and (less frequently) vasopressin and oxytocin from the posterior pituitary.

**Table 4.** Key genes described in combined pituitary hormone deficiencies involving luteinizing hormone/follicle-stimulating hormone. Sourced and adapted from (154, 174) and Genomics England Pituitary Hormone Deficiency panel (3.1)

Transcription factor of pituitary development	Inheritance	Hypothalamo-pituitary axis impairment	Potential phenotypic characteristics
*BRAF*	Autosomal dominant	CPHD	SOD, hypopituitarism, cardio-facio-cutaneous syndrome
*FOXA2*	Autosomal dominant	CPHD, congenital hyperinsulinism	Craniofacial dysmorphism, ocular abnormalities, cardiac, gastrointestinal, and genital anomalies
*GLI2*	Autosomal dominant, haplo-insufficiency	GH, CPHD	Holoprosencephaly, midline defect, ectopic pituitary lobe, polydactyly, Culler-Jones syndrome
*GLI3*	Autosomal dominant	GH, ACTH, CPHD	Greig cephalopolysyndactyly, Pallister-Hall syndrome, hypothalamic hamartoma, polydactyly
*HESX1*	Autosomal dominant or recessive	GH, LH, FSH, TSH, ACTH, CPHD	SOD PSIS: triad of aplasia or hypoplasia of pituitary stalk, hypoplasia of anterior pituitary gland, ectopic posterior pituitary lobe; hypoplasia of corpus callosum
*HID1*	Autosomal recessive	CPHD	Syndromic infantile encephalopathy, developmental and epileptic encephalopathy
*IGSF1*	X-linked dominant	TSH, CPHD	Testicular enlargement
*LHX3*	Autosomal recessive	GH, TSH, LH, FSH, ACTH, prolactin, CPHD	Pituitary hypoplasia or hyperplasia, abnormal head and neck rotation, spinal anomalies, hearing loss
*LHX4*	Autosomal dominant	GH, CPHD	PSIS: triad of aplasia or hypoplasia of pituitary stalk, hypoplasia of anterior pituitary gland, ectopic posterior pituitary lobe; corpus callosum hypoplasia or Chiari syndrome; respiratory distress
*OTX2*	Autosomal dominant	GH, CPHD	Ocular malformations; PSIS: triad of aplasia or hypoplasia of pituitary stalk, hypoplasia of anterior pituitary gland, ectopic posterior pituitary lobe; Chiari syndrome
*PITX2*	Autosomal dominant	GH, PRL	Axenfeld-Reiger syndrome type 1, anterior segment dysgenesis
*PNPLA6*	Autosomal recessive	CPHD	Boucher-Neuhauser syndrome, Oliver-McFarlane syndrome, spastic paraplegia
*POU1F1*	Autosomal dominant or recessive	GH, TSH, PRL, CPHD	Hypoplasia of pituitary gland
*PROP1*	Autosomal recessive	GH, TSH, PRL, LH, FSH, variable ACTH, CPHD	Hypoplasia of pituitary gland (potentially after transient pituitary hyperplasia)
*RNPC3*	Autosomal recessive	GH, TSH, CPHD	Pituitary hypoplasia, developmental delay, delayed puberty, congenital cataract
*SOX2*	Autosomal recessive	LH, FSH, variable GH, CPHD	Microphthalmia, intellectual disability, sensorineural hearing loss, esophageal atresia, micropenis
*SOX3*	X-chromosomal	GH, CPHD	PSIS: triad of aplasia or hypoplasia of pituitary stalk, hypoplasia of anterior pituitary gland, ectopic posterior pituitary lobe; intellectual disability, digital anomalies, micropenis
*TBC1D32*	Autosomal recessive	GH, TSH, LH, FSH, CPHD	Oro-facial-digital syndrome, craniofacial anomalies, hypopituitarism, anterior pituitary hypoplasia, ectopic posterior pituitary

Abbreviations: ACTH, adrenocorticotropin; CPHD, combined pituitary hormone deficiencies; FSH, follicle-stimulating hormone; GH, growth hormone; LH, luteinizing hormone; PRL, prolactin; PSIS, pituitary stalk interruption syndrome; SOD, septo-optic dysplasia; TSH, thyroid stimulating hormone (or thyrotropin).

A population study in Finland estimated the incidence of congenital CPHD at 1 in 16 000 (175). Of their cohort of 48 patients with CPHD, most (60.4%) were of idiopathic origin, 14.6% had a genetic diagnosis (due to defects in *PROP1*, *SOX3*, *SOX2*, *OTX2*, and *TBC1D32*), and the remainder (25%) had septo-optic dysplasia without a genetic diagnosis. Of these 48 patients, 8 male patients (16.7%) presented with a genital phenotype of micropenis and/or bilateral cryptorchidism; no female patients had an identifiable genital phenotype. Seven of these 8 patients were treated during mini-puberty, 5 with rFSH and testosterone (176) and 2 with only testosterone. All in the cohort who underwent brain magnetic resonance imaging (MRI) had abnormalities, of which 90% included classical findings such as pituitary stalk interruption syndrome (PSIS), with anterior pituitary hypoplasia, absent/ectopic posterior pituitary, and a hypoplastic infundibulum. Extrapituitary signs in patients with congenital CPHD included craniofacial, eye, genital, ear, and dental abnormalities, abnormalities of thermoregulation, musculoskeletal anomalies including arthrogryposis, and developmental delay or epilepsy (175, 176).

Hypogonadotropic hypogonadism is not universal among congenital CPHD, but neither is it uncommon. A study of 34 infants in Argentina with congenital hypopituitarism found that 19 had an abnormal HPG axis and CHH (177). The reason for the referral among male patients was in the majority due to hypoglycemia or prolonged jaundice due to cholestasis, but on detailed examination 15 out of 21 male patients were also identified to have abnormal external genitalia, with 12 out of 21 having both micropenis and cryptorchidism (unilateral or bilateral). After biochemical testing, 14 out of 21 had low LH and low testosterone (177). In summary, up to 70% of male infants with CPHD have congenital HPG axis disturbances and should therefore be screened to investigate for low concentrations of gonadotropins and sex steroids during the first months of life.

### Syndromic Conditions Associated With Gonadotropin-releasing Hormone Deficiency

At least 11 syndromes have been described in association with CHH and hence disordered mini-puberty (117). CHARGE syndrome is an autosomal dominant disorder associated with coloboma, heart defects, choanal atresia, reduced growth/development, and genital and ear anomalies (178). Mutations in the *CHD7* gene underlie around two-thirds of cases. Genital anomalies are most commonly cryptorchidism and micropenis in males, and authors have recommended measurement of LH and FSH in infancy to help establish the diagnosis (179). In a study of 32 children, 19 out of 20 males had either cryptorchidism or micropenis, 7 of 9 aged younger than 5 months had extremely low testosterone, and none of the girls older than 12 had progressed into spontaneous puberty (180). Of note, *CHD* mutations can cause CHH or Kallmann syndrome without other associated features of CHARGE syndrome.

Waardenburg syndrome is an auditory-pigmentary syndrome associated with changes to the pigment of the hair and eyes, distinctive facial features, as well as congenital sensorineural hearing loss and—in type 4—additional Hirschsprung disease (181). Waardenburg syndrome types 2 and 4 have been linked to mutations in *SOX10*, which encodes the sex-determining region Y-box 10 protein. *SOX10* has also been investigated in hypogonadotropic hypogonadism after it was noted that some patients with Waardenburg syndrome have agenesis of the olfactory bulb, seen in Kallmann syndrome (138). Subsequently, mutations in *SOX10* have been described in CHH, both anosmic and normosmic forms, and some have suggested that patients with *SOX10* mutations represent a phenotypic continuum across these two disorders. Some male patients have cryptorchidism, suggesting the potential for diagnosis in infancy and disordered mini-puberty (182, 183).

(Laurence-Moon-) Bardet-Biedl syndrome (BBS) is an autosomal recessive disorder with features including obesity, retinitis pigmentosa, polydactyly, intellectual disability, and hypogonadism (184). Hypogonadotropic hypogonadism has been described in males and females with this syndrome (185), although the prevalence is low (186), as well as hypergonadotropic hypogonadism (187). Males with BBS may have not only partially defective GnRH and or gonadotropin secretion, but also defects within the gonads. Individual case reports show evidence of a variably disordered spermatogenesis. Spontaneous paternity of men with BBS has been reported (188).

Males with Prader-Willi syndrome have more pronounced mixed central and primary gonadal defects underlying their hypogonadal phenotype (189). Almost all male patients with Prader-Willi have unilateral or bilateral cryptorchidism at birth, in addition to intellectual disability, morbid obesity, and additional pituitary hormone deficiencies (190).

Other genetic conditions with CHH commonly include other neurodevelopmental issues. Gordon-Holmes syndrome (191), Boucher-Neuhäuser syndrome (192), and Oliver-McFarlane syndrome represent a phenotypic cluster within a spectrum of neurodegenerative disorders. These syndromes may be caused by mutations in the *PNPLA6* gene; all are associated with hypogonadotropic hypogonadism.

Patients with *SOX11* syndrome have intellectual disability (40), patients with loss-of-function variants in *NLGN3* have developmental delay and autistic spectrum disorders (193), and those with cerebellar hypoplasia and intellectual disability are associated with recessive variants in the *PRDM13* gene (194).

Population studies also support the existence of common genetic determinants between neurodegenerative disorders and CHH (195).

### Effect of the Gene-Environment Interface on Mini-Puberty

It has been demonstrated that variations in progression through mini-puberty among healthy infants may be explained by environmental factors. Studies of monozygotic and dizygotic infant twins using quantitative genetic modeling have reported that most of the interindividual variability observed in salivary testosterone could be explained by environmental rather than genetic factors, both for males and females (196, 197). However, a study of cord blood from 58 same-sex twins found that genetic factors were a significant contributor to within-sex differences in progesterone and estradiol, although again not for testosterone, which was primarily explained by intrauterine environmental factors (198).

The influence of environmental toxins, known as endocrine-disrupting chemicals (EDCs), on the HPG axis has been much studied. In male infants, higher levels of in utero exposure to phthalates was shown to be associated with undermasculinization of the genitalia with reduced anogenital distance (199). Data from several animal models have demonstrated the effect of EDCs, including common compounds such as paracetamol, on testicular descent and function during gestation and in infancy (200). Effects on central elements of the HPG axis have also been shown, with exposure of female fetuses to arsenic leading to alterations in the expression of GnRH and LH, but also of upstream hypothalamic transcriptional regulation (201). Exposure during the mini-puberty period also represents a critical window for EDC exposure and immune regulation (202, 203).

While direct exposure prenatally or postnatally may be important, the effect of EDCs may also be cross-generational, with effects of exposure in pregnant rodents seen for 2 or more generations to come (204). Thus, EDCs may influence the reproductive axis through epigenetic mechanisms both within the gonad but also centrally via modulation of hypothalamic gene expression (205, 206).

### Mini-Puberty in Primary Gonadal Disorders: Disorders/Differences of Sexual Development and Cryptorchidism

Mini-puberty has been characterized in several primary gonadal disorders in the context of differences of sexual development (DSD), detailed description of which is beyond the scope of this review and has been previously expertly detailed (207-210).

#### Mini-puberty in differences of sexual development

The importance of mini-puberty in individuals with primary gonadal dysfunction is as a window of opportunity for its early diagnosis. This includes 46,XY conditions in which there is undervirilization at birth due to testicular endocrine dysfunction, such as partial LH receptor defects, partial defects in steroidogenesis (eg, 17ßHSD3 deficiency), partial androgen insensitivity, and 5-α-reductase-deficiency.

It also includes males or females with sex chromosome aneuploidies such as Klinefelter syndrome (classic 47,XXY or mosaic forms) with primary gonadal failure, XX testicular DSD, and (Ullrich-)Turner syndrome (classic 45X or mosaic forms including 46,XY/45X gonadal dysgenesis).

During mini-puberty basal concentrations of gonadotropins—particularly FSH—are likely to be *elevated* in males or females with DSDs and primary gonadal insufficiency. This represents a chance for early detection of gonadal dysfunction. Afterward, the HPG axis is silent until puberty. Therefore, after mini-puberty a biochemical diagnosis can no longer be made by measuring LH, FSH, testosterone, and estradiol alone. However, after mini-puberty the diagnosis of primary gonadal failure can still be indicated by low/undetectable AMH and inhibin B serum concentrations (208, 211-213).

#### Mini-puberty in males with isolated cryptorchidism

Isolated UDT at birth is not uncommon in infant boys. It affects up to 5% of term-born boys at birth, but spontaneous descent may occur in the first months after birth, leaving 1% of male infants with UDT by the end of the first year of life (214-216). In a large cohort of boys with UDT of unknown origin, relatively low serum testosterone levels were observed during mini-puberty compared to those of healthy controls; low testosterone concentrations were not accompanied by appropriately elevated LH levels. A relative FSH deficiency was also observed during mini-puberty in this cohort. After mini-puberty, the concentrations of the Sertoli cell marker AMH fell more sharply than those of controls (28).

In a separate study, cord blood INSL3 concentrations were significantly lower in boys with cryptorchidism than in controls, and in those who are persistently cryptorchid at 3 months, an increased LH to INSL3 ratio has been observed (25). A significant positive correlation was not found between INSL3 and LH and testosterone in boys with cryptorchidism, such as is seen in healthy boys. Thus, both aforementioned studies could suggest a disruption of the HPG axis during mini-puberty (25).

In adulthood, volumes of formerly UDT are on average lower, and fertility is reduced due to markedly impaired spermatogenesis, with rates of 70% to 90% oligozoospermia or azoospermia (217, 218), and nonsuccessfully attempted paternity in 45% (219). Thus, in boys with UDT, relative or absolute LH/FSH deficiency in mini-puberty could theoretically contribute to later fertility impairment. Moreover, there are observations of germ cell loss taking place in the cryptorchid testis, which is proportional to the duration of the condition (140). In boys with a history of UDT, the tumor risk is elevated 3- to 8- fold, dependent on the time at which correction of testicular position is achieved (220, 221). Therefore, delay in medical or surgical treatment to bring the testes into the scrotum is suspected to further affect testicular function.

Gonadotropins (hCG and nasal GnRH) have been used to induce testicular descent, which is successful in around 20% (222). Central hormone replacement been shown to increase the number of Ad spermatogonia seen on testicular biopsy (223).

## Consequences of Disordered Mini-puberty

### Phenotype in Infancy in Congenital Hypogonadotropic Hypogonadism as Compared to Healthy Infants

As described in detail earlier, disorders with deficient HPG function will result in a diminished or abolished mini-puberty. In male infants this early-life hypogonadism affects somatic development, manifesting with a variable phenotypic spectrum including reduced penile growth, an underdeveloped scrotum, and hypoplastic UDT (119). These clinical features are apparent shortly after birth, reflecting the understimulation of the genitalia during later gestation.

#### Cryptorchidism

Patients with bilateral UDT are particularly at risk of having an organic underlying cause and thus must be identified and referred early. As described earlier, the prevalence of cryptorchidism at birth in the general population is up to 5% (214-216), but among males with CHH it is up to 50%. Of note, while non-CHH infants with cryptorchidism may experience spontaneous testicular descent in the first 3 to 6 months of life, this is not the case in individuals with severe GnRH deficiency, making bilateral cryptorchidism after age 12 weeks an even more relevant diagnostic feature (215, 216, 224).

#### Micropenis

The frequency of micropenis in the hypogonadotropic hypogonadism cohort is also estimated to be much higher (>20% (165)) than in the general population (0.015%-0.3% (225, 226)). Micropenis is classically defined as a stretched penile length 2 or more SD smaller than the mean for age (approximate lower limit of normal for newborn <2.5cm, see Table 2). In male infants with CHH the phallus is often not only short but also of reduced girth. The features of micropenis alongside UDT may be accompanied by absent erections on diaper change.

If hormones are measured in the first months of life, low serum gonadotropin and testosterone concentrations are found, and this is indicative of hypothalamic-pituitary deficiency during the second wave of physiologic HPG axis action (see Section Approach to clinical assessment in phenotypic males) (159, 227, 228).

#### Nonreproductive features

The classic nonreproductive “red flag” phenotypes in males with underlying CHH that may be detectable at or after birth include cleft lip or palate, syndactyly or skeletal abnormalities, ear malformations, pits, or tags, and hearing impairment. Hearing impairment identified via an automated optoacoustic emissions test has a prevalence of 6% in CHH infants compared with a UK birth prevalence of 0.12% (229).

#### Linear growth and muscle mass

The neonatal androgen surge is furthermore associated with skeletal growth, with healthy male infants demonstrating increased linear growth velocity compared to female infants, which correlated with testosterone concentrations (230). In contrast, male infants with CHH display stunted linear growth during mini-puberty (231), with reduced height SD scores most marked at 3 to 6 months of life. However, this may be unrecognizable in the routine clinical setting and may not be relevant for later attainment of a height within the mid-parental target height.

Testosterone may have an effect on muscle mass in infancy by inducing greater total mass and lower fat mass due to the anabolic effects of androgens, which is shown through studies conducting comparisons of female and male infants (232). However, this is also difficult to detect in the routine clinical setting.

### Consequences for Mid-Childhood

#### Growth and body composition

Several studies have described the consequences of variations in mini-puberty for later childhood, even among apparently healthy infants, and especially for boys. However, evidence of a definitive effect of male CHH on phenotype in mid-childhood is scarce. In a study of 35 healthy babies, male and female, higher testosterone concentrations at age 8 weeks had a significant negative effect on body mass index up to age 3 years, triceps skinfold fitness up to 4.5 years, and body weight up to age 6 years, while LH concentrations had a negative effect on body weight and body mass index up to 6 years (50). However, there was no persistent correlation between hormone concentrations and anthrometric measurements in girls over this period. As linear growth in mid-childhood is mainly driven by the growth hormone–insulin-like growth factor-1 axis, individuals with isolated gonadotropin deficiency experience similar growth patterns to healthy children during this period.

#### Neurobehavior

Other studies have described a neurobehavioral role for mini-puberty. In a study of 22 boys and 26 girls, urine sampling for testosterone from 0 to 6 months was correlated with sex-typed behavior using the Pre-School Activities Inventory (PSAI) at 14 months (233). In a separate study of 81 healthy boys aged 3 to 4 years using the PSAI, prenatal androgen exposure, as reflected by anogenital distance, and postnatal androgen exposure as reflected by penile growth 0 to 3 months, were significantly associated with increased masculine behavior (234). However, a causal relationship for testosterone secretion during mini-puberty and neurobehavior cannot be inferred from these two small observational studies.

#### Language development

Mini-puberty has also been shown to be important for early language development. In a study of 16 healthy term infants (male and female) in Germany, estradiol concentration at 4 weeks (positive association) and testosterone concentration at 20 weeks (negative association) were significant predictors of infant articulatory skills at 20 weeks, with high effect sizes for both in hierarchical multiple regression analyses (235). Similarly, salivary testosterone collected at 4 to 14 weeks from 78 infants (male and female) negatively predicted (both in boys and girls) parent-reported expressive vocabulary scores at age 18 to 30 months, and testosterone remained a mediator between sex and scores even when other predictor variables were adjusted for (236). However, these associations are likely to also be affected by differences in sex chromosomal genes and their expression in boys and girls.

### Consequences for Adolescent Puberty

#### Pubertal development

In patients for whom disordered mini-puberty is reflective of HPG axis inactivation, the primary presentation in adolescence is of delayed, arrested, or absent puberty. Pubertal onset delay is defined by the absence of the first signs of puberty (Tanner stage B2 in girls and testicular development to size 4 mL measured by Prader orchidometry in boys) by 2 to 2.5 SD above the mean age of pubertal onset, ordinarily by age 13 in girls and age 14 in boys (237). Delayed progression through puberty may be defined by the use of puberty nomograms (238), or alternatively by nonaccomplishment of full pubertal maturation 5.5 years after pubertal onset.

Around 2% of adolescents experience pubertal delay. Of all boys with pubertal delay, constitutional delay of growth and puberty (CDGP) is present in 60%, while hypogonadotropic hypogonadism is identified as the etiology in 7% in boys (239) and around 10% to 12% of all patients (122, 240, 241). However, these proportions do not hold for older adolescents or young adults, in whom CDGP is the much rarer condition.

#### Hypogonadism

In untreated adolescent and adult males with CHH, low concentrations of testosterone due to insufficient LH stimulation of the Leydig cells are typical (7). The reduced Sertoli cell pool, reflected by low inhibin B concentrations, is already present before adolescence. Low inhibin B levels persist as compared to healthy pubertal males, as Sertoli cells do not expand nor mature; thus, the typical increase in testicular volume during puberty does not occur (242-244). In severe cases, without gonadotropin therapy, testicular volumes will remain very small. In those with pubertal arrest, there will be a slight increase in testicular volumes at the beginning of puberty, but with no further testis growth thereafter. Likewise, partial virilization is possible in less severe forms of CHH, with decompensation of endocrine testicular function at a variable time point during adolescence or adulthood, most likely at the beginning of puberty. In any case, spermatogenesis will not occur, with very rare exceptions. One exception is that of “Pasqualini syndrome,” which results from isolated failure of LH activity due to mutations in the *LHB* gene, thus giving rise to the phenotype of a “fertile eunuch” (245).

Therapeutic modalities for patients with CHH will be explored in subsequent sections. However, it should be noted that while induction of adolescent puberty is successful with the use of gonadotropins in male adolescents with CHH, induction of spermatogenesis is less likely to be fully successful in those with severe CHH, showing signs of disordered mini-puberty (lower baseline testicular volumes, a history of UDT, and micropenis). These patients have been shown to have lower final testicular volumes and smaller penile size despite adequate gonadotropin replacement during adolescence, than those patients with hypogonadotropic hypogonadism who do not display these severe features at birth. In patients with severe CHH, sperm counts in semen after 3 years of combined hCG/rFSH treatment remain well below the lower limit of the normal range (139, 166, 246-248). This is why, especially in these males with severe CHH, early recognition of central hormone deficiency is essential to allow for earlier adequate hormone replacement during mini-puberty.

#### Longitudinal growth

Untreated boys with CHH will also have slower height velocity than expected for age by adolescence due to a delayed or absent sex steroid–mediated pubertal growth spurt. Historically, men with CHH were expected to be tall for mid-parental height as hypogonadism was treated late, which led to delayed epiphyseal fusion. However, with timely treatment, height is expected to be in line with parental target height expectations (231, 249, 250).

#### Psychological development

**Figure 3.**
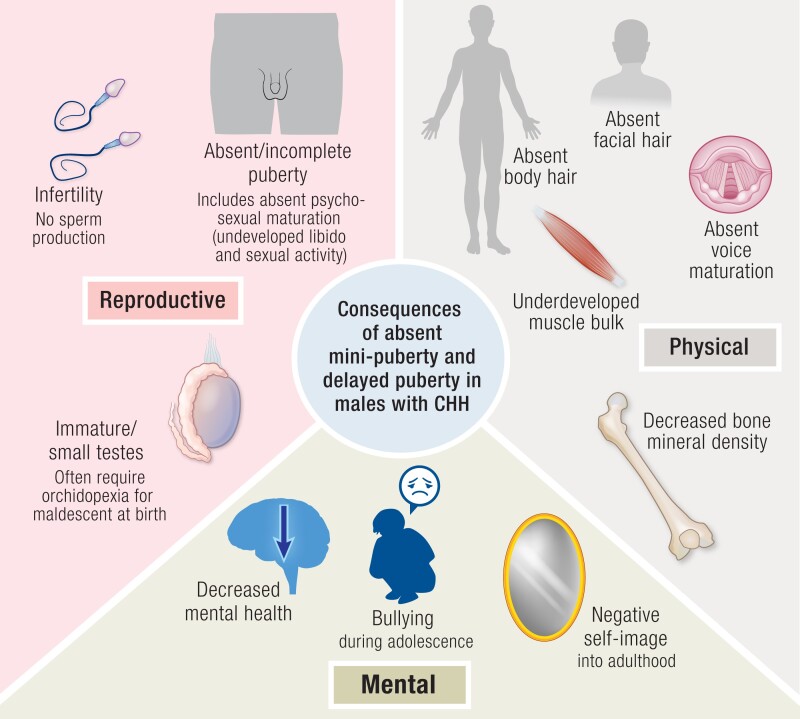
Long-term consequences of absent mini-puberty and delayed puberty, with late or nonphysiological therapeutic intervention in males with congenital hypogonadotropic hypogonadism (CHH).

By the time of adolescence, another key consequence of early central hypogonadotropic failure is psychological (Fig. 3). In a study including 60 male adolescents with various causes of early childhood-onset hypogonadotropic hypogonadism, those treated with testosterone for absent puberty scored much higher in health-associated problem scores and were less satisfied with their masculinity than those who had received gonadotropins replacement primarily during adolescence (251). In a study of psychosexual development in men with CHH who were treated with testosterone until their desired fertility, 93% reported experiencing concerns about their body image, feeling embarrassed or ashamed by their bodies, and avoiding undressing in public spaces (252). In this cohort study, 73% described having been teased because of their condition, and other surveys have also described participants experiencing bullying during adolescence (157). Also, negative self-image has been found to be associated with high rates of depressive symptoms both in men and women with hypogonadotropic hypogonadism (156, 253, 254).

Individuals with CHH are also less likely to form relationships, particularly in adolescence and young adulthood. Those individuals with more severe GnRH deficiency reported worse outcomes for sexual health (255). The reasons for these long-term effects on psychosexual development are likely to be multifaceted, but may be in part due to deficiency of appropriate HPG activity during mini-puberty (256).

Thus, conventional treatment with testosterone, and later with central hormone replacement for those who desire paternity, does not ameliorate all negative psychological sequelae. However, one study of health-related quality of life in 31 men who received gonadotropin therapy (hCG and rFSH) over 2 years demonstrated significant increases in almost all domains, including in role-emotional and mental health, with greater increases in those patients with improvements in spermatogenesis (257).

### Later Consequences: Fertility, Health, and Cognition in Adulthood

#### Fertility and sexual function

Fertility is one of the key outcomes important to young adults with CHH and thus disordered mini-puberty and puberty. In males, there is evidence that mini-puberty has lasting consequences for fertility even among healthy infants.

In a population cohort study, 259 boys were examined clinically and biochemically at age 3 months and again at age 18 to 20 years (258), with a positive association seen between testosterone at 3 months and adult total sperm count. The median (interquartile range) total sperm count at 18-20 years in those with the lowest tertile of testosterone in infancy was 84 (54-138) million spermatozoa, compared to 193 (56-287) million spermatozoa for those in the upper tertile, and other reproductive hormones also showed good correlation between infancy and adulthood.

Additionally, cryptorchidism, even among men who are otherwise healthy, is associated with impaired semen quality (259). Testicular tissue biopsies from patients with cryptorchidism and absent mini-puberty show lack of transformation of gonocytes into Ad spermatogonia cells, which are involved in maintaining stem cells for later spermatogenesis (260). It has been shown that apoptosis of germ cells is greater in infants with low FSH than in those with normal or high FSH (261). Therefore, it is likely that attempts to induce spermatogenesis in CHH men who have not experienced mini-puberty and have poor testicular volume are therefore less likely to be successful than in those who have (165, 262).

Fertility difficulties due to physiological and anatomical differences are exacerbated by low-self-image, which leads to an increase in the proportion of men with CHH who have never been sexually active (with a partner) compared to the general population (26% vs 5.4%; *P* < .001) (252).

Despite these negative consequences for fertility, it may be of interest to patients that, despite testicular pathology, testicular cancer is exceedingly rare among men with CHH, leading some to suggest that CHH may even be protective against testicular cancer in later life (263).

In women with CHH, as previously described, the long-term functional significance of disordered mini-puberty is thought to have less effect on fertility than in men because women in general respond well to central hormone replacement in adulthood with immediate restoration of fertility, provided that uterine growth has previously been initiated by estrogen substitution. This is also reflected by the observation that women with CHH are more likely to have been in a relationship than men with CHH, and more likely to have biological children (80% vs 28%; *P* < .01) (157). However, other consequences for sexual function have been described, including reduced lubrication during arousal and lower orgasm scores (264). The latter could be the consequences of insufficient replacement of ovarian estrogens and/or androgens in certain therapeutic regimens.

#### Bone health

There are additional lifelong consequences for patients with CHH in terms of impaired bone health. Estrogen is the key sex steroid for attainment of peak bone mass both for men and women and is low both in men and women with CHH (265). In a study of 14 adolescent girls with hypogonadotropic hypogonadism, a low bone mineral density (BMD) *z* score (−2 SD) was seen for 10 out of 14 patients (71.4%), and there was a strong negative correlation between chronologic age and height-adjusted BMD *z* score measured from the spine (266), possibly as a consequence of delay in starting hormone replacement.

Androgen signaling and testosterone are also involved, but to a different extent, in bone development and bone mass (267). Low serum testosterone and consequently low serum estradiol concentrations in men with CHH increases the risk of osteoporosis and low-impact fractures (265). A study of 51 men with CHH showed significant impairments in BMD and bone microarchitecture compared to controls, but early treatment (before age 19 years) had a positive protective effect on trabecular outcomes (268).

#### Neurodevelopment

GnRH cells are present in several extrahypothalamic brain regions in addition to the hypothalamus (269). Patients with hypogonadotropic hypogonadism have been shown in one epidemiological study to have an increased risk of diagnosis of neurodevelopmental disorders including autism, attention deficit hyperactivity disorder, as well as intellectual disability, as compared to controls (195). Other studies in CHH have also described cognitive problems, including impaired spatial ability correlating with testicular volume (270).

In a study of 34 newly diagnosed and untreated men with hypogonadotropic hypogonadism (mean age 29.1 ± 10.5 years), significantly worse executive function, attention, visual scanning, and psychomotor speed were observed compared to healthy controls (271). This suggests that disordered central HPG axis function, including mini-puberty, may be linked to long-term neurodevelopmental outcomes. The common co-occurrence of CHH with other neurological signs, including synkinesia and ataxia, may suggest that the role of mini-puberty for neurodevelopment should be further investigated (272, 273).

## Identification and Workup of an Infant With Disordered Early Hypothalamic-Pituitary-Gonadal Axis Activation

### Barriers to Diagnosis of Disorders of Mini-Puberty, and Pathways to Optimization

A high proportion of patients with disordered mini-puberty may not be identified at birth or in infancy. This may be due to a lack of clinical signs, or incorrect interpretation of these signs by the clinician. Ultimately, this missed opportunity for diagnosis in infancy results in most cases of CHH being diagnosed due to delayed adolescent puberty. As a rare condition, with only between 26 to 88 boys expected to be born with CHH each year in the United Kingdom or Germany, and as few as 4 to 14 boys in Sweden or Switzerland, it is not a presentation with which most general practitioners are very familiar.

While it is not possible to screen for absent mini-puberty routinely in healthy infants, a targeted approach to either evaluate mini-puberty or to refer for evaluation male infants with maldescended testes or micropenis to a pediatric endocrinology unit, when these features are detected at routine baby checks by general practitioners, as well at pediatric, pediatric surgical and neonatal pediatric review, is recommended (274).

Additionally, infants diagnosed with CPHD, who most commonly present with hypoglycemia due to ACTH deficiency with hypocortisolemia or with prolonged jaundice due to congenital secondary hypothyroidism, with or without UDT and micropenis, and who are under the care of inpatient pediatric neonatal or endocrine services, could be assessed biochemically for CHH and thus can be diagnosed before or within the mini-puberty window.

Increasingly, fertility services can also refer for assessment and management infants born to parents with CHH. The majority of patients with CHH require fertility therapy to have children, and thus could be highly motivated to preserve the reproductive capacity of their own children. Thus, infants could be referred to pediatric endocrine services also via this pathway.

### Approach to Clinical Assessment in Phenotypic Males

**Figure 4.**
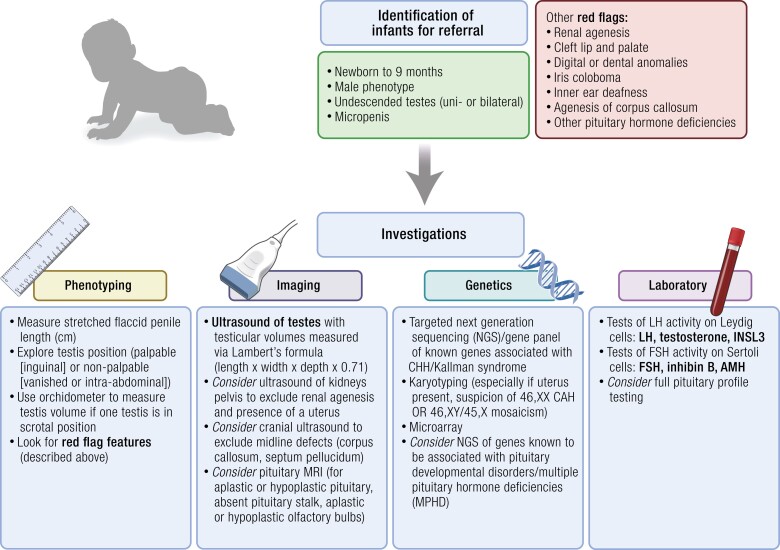
Identification of male infants with suspected congenital hypogonadotropic hypogonadism (CHH): investigations to confirm the diagnosis, exclude differences of sexual development, and detect associated malformations.

Patients with genital abnormalities including micropenis and cryptorchidism (in particular bilateral cryptorchidism) will in many countries warrant referral to pediatric endocrinology as part of DSD pathways (275). These signs should be identified as part of the routine newborn examination (276). Increased awareness is required that the presence of these signs at birth can be indicative of absent activity of the antenatal HPG axis and potentially CHH or other associated disorders. Fig. 4 provides guidance as to how to identify male infants with CHH, which investigations are indicated to confirm the diagnosis during mini-puberty, how to exclude DSD, and how to detect associated malformations.

If cryptorchidism is identified, the position and size of the testes will need to be confirmed on ultrasound. Micropenis should be determined by measurement of stretched penile length, as compared to age-appropriate normal ranges (see Table 2). Micropenis that is present as an isolated feature or found in combination with other urogenital anomalies, particularly hypospadias, may point toward a wide range of DSD conditions. Hypospadias is not a typical feature of isolated CHH.

While the key red flags for identification of CHH in boys that are apparent shortly after birth are cryptorchidism and micropenis, as well as absent erections on diaper change, other features may lead to consideration of this condition. These include those signs found among males with hypogonadotropic hypogonadism in a minority of patients, such as renal agenesis, cleft lip and palate, hypodontia, congenital ptosis, hearing loss, ear deformities, skeletal and digit abnormalities (alongside hyposmia, which can be recognized following radiological investigation or when communication is possible) (153). Evidence of deficiency of other anterior pituitary hormones, such as neonatal hypoglycemia, jaundice, or dysmorphic features, may point to CPHD.

Other clinical features used by some centers to assist in clinical evaluation, especially if DSD is suspected, include anogenital distance and digit length ratio, which are thought to reflect fetal androgen exposure, whereas, conversely, penile length is affected both by prenatal and postnatal androgen exposure (277). Anogenital distance has been shown to be significantly reduced in infants with hypospadias (278), and moderately reduced in those with cryptorchidism (279). However, to our knowledge, this has not been quantified in a cohort with CHH, so it should not be relied on as part of a diagnosis. Reference values have been quantified in a cohort of predominantly White children (280). The 2:4 digit ratio is the ratio of the length between the second and fourth fingers; males tend to have longer fourth fingers than females. However, there is conflicting evidence about its reliability as a reflection of fetal androgen exposure and again should not be relied on routinely as part of diagnostic evaluation (281).

For patients referred to pediatric endocrinology with micropenis or cryptorchidism, measurement of serum testosterone, estradiol, and gonadotropins are recommended. Precise timing of these tests is important, ideally at least 2 separate measurements within 4 to 26 weeks of life, to target the known window of gonadotropin activation during mini-puberty (274). While undetectable or very low concentrations of LH and FSH, alongside low or undetectable sex steroid concentrations suggest the diagnosis of CHH (120), evidence of elevated FSH and LH are indicative of primary gonadal insufficiency and should be investigated according to DSD guidelines (275).

Due to their status as a reflection of Sertoli cell population, inhibin B, as well as AMH, are typically low both in CHH and in primary gonadal disorders, but are recommended to provide information on testes development (275, 282). However, inhibin B and AMH may not be available at all centers and are more costly than gonadotropin or sex steroid assays. INSL3 measurement is not available as a clinical test in most units, but may be available as part of a research study. If CHH is suspected, a full clinical evaluation will be required, including assessment for growth, midline defects, and other pituitary hormone deficiencies (ACTH, thyroid function, insulin-like growth factor-1 (IGF-1), prolactin) (120), as well as MRI of the brain including pituitary and olfactory bulbs (or cranial ultrasound if MRI is not available) and renal ultrasound. GnRH stimulation testing is not widely used during infancy and has not been validated for assessment of mini-puberty. However, it is used in some centers in cases where basal gonadotropin concentrations are in the intermediate range and the diagnosis is unclear, with diagnostic thresholds for peak LH and FSH as per in adolescence (119).

In patients with associated CPHD, radiological identification (by MRI) can aid early identification of disturbances of central HPG axis function. These include pituitary hypoplasia or aplasia, with or without absent pituitary stalk or an ectopic posterior pituitary as part of pituitary stalk interruption syndrome (PSIS), or midline defects, such as an underdevelopment of the corpus callosum or an absent septum pellucidum (175).

While karyotyping is relevant if there is a clinical suspicion of 46,XX congenital adrenal hyperplasia (CAH) or chromosomal mosaicism, further genetic testing may also be appropriate at this point, primarily to help confirm the diagnosis of CHH or Kallmann syndrome, but may also help to guide therapies. Patients with GnRH receptor mutations have demonstrated a resistant response to pulsatile GnRH administration, and thus gonadotropin replacement is more appropriate in such cases (283). As described earlier, many countries have now panel or virtual panel exome testing for CHH, with the number of genes tested varying widely between different centers. Targeted NGS may also be used for identification of the genetic origin of male infants with CPHD. The use of microarray testing for copy number variation is generally reserved for cases with syndromic features or associated dysmorphology or learning difficulties.

### Approach to Clinical Assessment in Phenotypic Females

The investigation of girls at birth is more challenging, as external features are not different in girls with CHH and thus the condition goes undetected unless biochemical and/or genetic testing is undertaken. Females are much more likely to be identified if they have hypogonadotropic hypogonadism in the context of an overarching disorder presenting with midline defects and other pituitary hormone deficiencies, which may produce more obvious signs. Alternatively, patients may be investigated due to a parent or sibling having CHH or a related condition. However, whether this is ethically appropriate is questionable in a cohort where no treatment is currently recommended before adolescence.

## Current Treatment Options for Disordered Mini-Puberty

### Pharmacologic Modalities in Mini-Puberty

Current interventions applied in infancy for male infants with CHH include topical or intramuscular application of testosterone, in particular for the treatment of micropenis. In males, testosterone has been used for several decades. Studies since the 1970s and 1980s have described the use of topical testosterone to increase the length and girth of the penis in patients with micropenis (284, 285). A 2001 study described the administration of 0.2 g of 5% testosterone ointment daily for 30 days to 50 boys with micropenis (aged 5 months to 8 years), leading to a significant increase in penile length (286).

An alternative modality is to give a 3-month course of intramuscular testosterone, such as intramuscular injection of 25 mg of testosterone cypionate or enanthate every 3 weeks for 3 months (287-289). Studies have also described topical application of DHT in gel form for children with micropenis, and 1 study described 61% of a sample of 23 children achieving a stretched penile length within −2.5 SD of the population mean after treatment for 6 months with DHT gel (290).

The Consensus Statement of the European consortium studying GnRH biology, published in 2015, formally recommends treatment of micropenis with a short-term course of low-dose testosterone (120). However, testosterone and DHT do not promote the proliferation of Sertoli cells or testicular growth and development, neither does it induce descent of maldescended testes (291).

### Surgical Management of Undescended Testes

Orchidopexy, a surgical procedure whereby UDT are moved and surgically fixed in the scrotum, with or without pretreatment with short-term hCG or nasal GnRH, has been the recommended treatment modality for UDT, including in those with CHH (292-294). Current therapeutic standards in Europe are inconsistent, but most recommendations aim at normalizing the position of the testes during the second half of the first year of life (295). The Consensus Statement of the European consortium studying GnRH biology recommends surgical correction of cryptorchidism between age 6 and 12 months (120).

In summary, the current standard of care for neonatal UDT does not consider the underlying pathophysiology. The timing of therapy and duration of GnRH nasal spray therapy is insufficient to mimic mini-puberty, and the protocols using hCG injections lack FSH and are also given for only 3 weeks. Current treatment strategies with hormones (nasal GnRH for 4 weeks or weekly intramuscular injections of hCG for 3 weeks) are successful in promoting testicular descent in 20% to 25% (296, 297) of patients; however, reascension rates of 25% of these lead to a final success rate of only 15% (298). Surgical orchidopexy has a success rate of around 90% and is therefore the preferred first-line treatment in many countries. However, this may potentially cause trauma to the testes and in some cases (∼2%), testicular atrophy (299). This risk of atrophy is highest in male infants with an intra-abdominal position of the testes. A key difference between orchidopexy in CHH vs isolated cryptorchidism is the nature of the short spermatic cord, which thereby requires greater surgical mobilization. In those with bilateral cryptorchidism due to CHH, surgery alone is also not enough to increase the potential for fertility (300).

### Limitations of Current Therapies

Historically, the key therapeutic focus for patients with CHH has been the induction of puberty in adolescence. This has typically involved the use of sex steroids, testosterone in boys and estradiol (followed by the introduction of progesterone) in girls. For boys, while testosterone induces many signs of puberty including induction and maintenance of secondary sexual development, optimization of bone health, muscle strength, and libido (301), it will not promote testis growth nor potential for spermatogenesis when given in isolation (166). This has led to increasing use of gonadotropins for the induction of puberty because of their role in inducing spermatogenesis (162, 302-305).

In a parallel fashion, increasing consideration has been given to the use of gonadotropins in male infants to recapitulate mini-puberty. The goal of treatment in patients with disordered mini-puberty is to restore the normal HPG activation during this developmental window, and thus to prepare for later adolescent puberty, fertility, sexual function, and to preserve bone health and mental health (176, 228, 306-310).

## Gonadotropin Therapy for Replacement of Mini-Puberty in Males

### Biological Basis for Gonadotropin Replacement in Male Infants

As use of gonadotropins for adolescent puberty in hypogonadotropic hypogonadism has become more common, it has become clear that a key predictor of successful outcome is initial testicular size. A larger basal testicular size is well documented to lead to higher rates of induction of spermatogenesis in puberty for these patients (160-163). The typical treatment regimen for adolescent or adult patients with CHH involves a combination of hCG and rFSH (166). In patients with severe CHH and low testes volume, pretreatment with rFSH prior to combined gonadotropin therapy or prior to GnRH pump therapy has been suggested to improve outcomes including spermatogenesis (120, 150). This pretreatment with rFSH in adolescence or adulthood essentially aims to recapitulate the Sertoli cell expansion that occurs in male infants. Thus, a more physiological approach would logically be to try to achieve this through induction of mini-puberty at the correct developmental time point. The potential increase in testicular size that could occur after treatment with gonadotropins in infancy can also reduce the technical challenges of performing orchidopexy. Consequently, this observation has driven interest in the application of gonadotropin replacement in infancy, to induce mini-puberty, and to improve reproductive capacity in adulthood (210, 311-313).

### Previous Evidence on Safety, Efficacy, and Outcomes

The first case report of treatment with gonadotropins to induce mini-puberty was published in 2002. An infant with CHH was treated with recombinant human LH and FSH twice weekly from 7.9 months until around 13.7 months, resulting in an increase in testicular volume by 170%, an increase in penile length from 1.6 to 2.4 cm, and inhibin B concentrations being achieved within the normal range (306).

**Figure 5.**
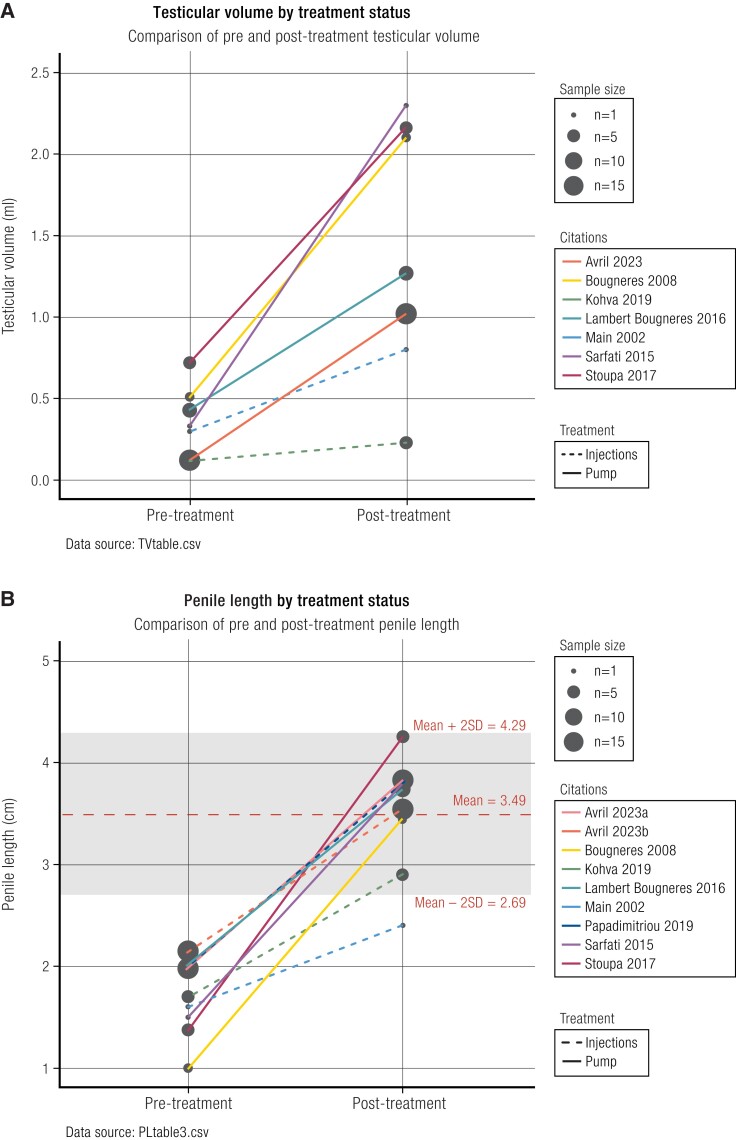
A, Change in testicular volume (pre to post treatment) with gonadotropin or gonadotropin-releasing hormone (GnRH) therapy used to replace mini-puberty in male infants with congenital hypogonadotropic hypogonadism (CHH), according to published case series (until June 2023). Size of study participants in the respective studies are illustrated by the size of the black circle (see sample size legend). Normal range varies by ethnicity and method of measurement (see Table 3), therefore no normal range has been plotted. For one study (Papadimitriou et al (315)) pretreatment values were not available; for one subgroup (Avril et al (310)) injection subgroup data were not available; both not plotted. All values represent means or medians measured with ultrasound (where specified). B, Change in average penile length (from pre to post treatment) with gonadotropin or GnRH therapy used to replace mini-puberty in male infants with CHH, according to published case series (until June 2023). Size of study participants is shown by the size of the black circle (see sample size legend). Stretched penile length of healthy male infants is shown as mean (horizontal dashed line) ± 2 SD (gray-shaded area), results from Boas et al (51), the largest study to date (see also in Table 3).

Subsequently, more case reports and case series have been described of successful treatment of these patients using gonadotropins to induce both biochemical and clinical signs of successful mini-puberty (Table 5). Three studies described the administration of a continuous LH and FSH infusion via subcutaneous pump for 6 months in 11 infants (228, 307, 309), and 1 further study described the same regimen for between 3 to 6 months in 5 infants (308). Although variable outcomes were assessed, all studies assessing each outcome reported that this regimen led to increased circulating concentrations of FSH, LH, inhibin B, and testosterone after short-term follow-up. AMH was increased in 1 study for 7 out of 10 patients (228). Clinically, all studies reported increased stretched penile length, increased testicular volumes, and testicular descent, illustrated in Fig. 5A and 5B. In Fig. 5A average testicular volumes clearly increased for all studies, except for one, which used the most restrictive therapeutic approach, and Fig. 5B shows that average penile lengths increased to within 2 SD of the population mean in all but 1 case report. One study reported sustained normal testicular volume of 0.8 mL for 1 patient at 1 year after end of treatment (309).

**Table 5.** Studies describing gonadotropin therapy for induction of mini-puberty in males with hypogonadotropic hypogonadism

Citation	Sample size	Population	Age at treatment initiation	Intervention	Biochemical outcome	Short-term clinical outcome	Long-term clinical outcome
Main et al 2002 (306)	1	n = 1 hypogonadotropic hypogonadism	7.9 mo	Twice-weekly recombinant LH injections from 7.9 to 12.2 mo Twice-weekly recombinant FSH injections from 7.9 to 13.7 mo	Increased LH, FSH, estradiol, and inhibin B to within normal range Testosterone remained undetectable	Increased testicular volume by 170% Increased stretched penile length from 1.6 to 2.4 cm	Not reported
Bougnères et al 2008 (307)	2	n = 1 hypopituitarism n = 1 hypogonadotropic hypogonadism	2-5 mo	Continuous recombinant LH and FSH infusion via SC pump for 6 mo	Increased LH, FSH, and inhibin B to within normal range Increased testosterone	Increased testicular volume from 0.45 to 0.57 mL at birth to 2.10 mL at 7 mo Increased stretched penile length for both patients	Not reported
Sarfati et al 2015 (309)	1	n = 1 KS	1 mo	Continuous recombinant LH and FSH infusion via SC pump for 6 mo	Not reported	Increased testicular volume from 0.33 to 2.3 mL Increased penile length from 1.5 to 3.8 cm	Testicular volume normal (0.8 mL) at 1 y after end of therapy
Lambert and Bougnères 2016 (228)	8	n = 5 isolated hypogonadotropic hypogonadism n = 3 combined pituitary hormonal defects	Mean 6.03 mo	Continuous recombinant LH and FSH infusion via SC pump for mean 6 mo	Increased LH, FSH, testosterone, inhibin B for all patients; increased AMH for 7 of 10 patients	Increased testicular volume in those with palpable testes Testicular descent in all patients Increased stretched penile length from mean 2.02 cm to 3.74 cm	Not reported
Stoupa et al 2017 (308)	5	n = 4 CHH n = 1 panhypopituitarism	Mean 4.2 mo	Continuous recombinant LH and FSH infusion via SC pump for 3-6 mo	Increased LH, FSH, inhibin B, AMH, testosterone	Increased testicular length and volume Increased stretched penile length from mean 1.38 cm to 4.26 cm	Not reported
Papadimitriou et al 2019 (315)	10	n = 10 bilateral cryptorchidism in intra-abdominal or inguinal position and micropenis, of which: n = 5 KS n = 1 syndromic features and neurodevelopmental delay *n* = 1 idiopathic hypogonadotropic hypogonadism n = 1 CHARGE syndrome n = 2 SOD n = 1 congenital panhypopituitarism	Median 4.2 mo	Daily injections of combination recombinant LH and FSH for 3 mo	Increased LH, FSH, inhibin B, AMH, testosterone to within or above normal	Testes measured 1.5 mL Testicular descent in all patients, 2 reascended and required orchidopexy Increased stretched penile length from median 2 cm to 3.8 cm	After 3-10 y of follow-up, all testes remained in scrotal position, with regression in size to 1.0 mL (0.5-2.0 mL); no spontaneous pubertal maturation yet
Kohva et al 2019 (176)	5	n = 4 combined pituitary hormone deficiency n = 1 CHARGE syndrome	Mean 2.5 mo	2 or 3 times weekly recombinant FSH injections for 3-4 mo Once monthly injection of testosterone for 3 mo	Increased FSH, inhibin B during treatment	Testicular volume increased for some patients; 2 cases of ascent of testes post treatment Increased penile length from mean 1.7 to 2.9 cm	Inhibin B concentration after 11.2 ± 1.5 y did not significantly differ from untreated CHH controls
Avril et al 2023 (310)	35	n = 35 CHH	Mean 5.1 mo (pump group), mean 13 mo (infusion group)	n = 18 continuous administration of recombinant LH and FSH by pump for 6 mo, n = 17 multiweekly SC injections of recombinant hCG and FSH for 3 mo	Increased FSH, inhibin B, AMH in both groups Significantly greater increase in testosterone in injection group compared to pump group	Increased testicular volume Improved testicular descent Increased penile width—all of above for both groups Significantly greater rate of increase in penile length in injection group vs pump group	Not reported

Abbreviations: AMH, antimüllerian hormone; CHH, congenital hypogonadotropic hypogonadism; FSH, follicle-stimulating hormone; hCG, human chorionic gonadotropin; KS, Kallman syndrome; LH, luteinizing hormone; SC, subcutaneous; SOD, septo-optic dysplasia.

Only one study has conducted a comparison between different regimes, Avril et al (2023), which compared continuous recombinant LH and FSH via pump against use of subcutaneous injections of recombinant hCG and FSH (310). The authors described a significantly greater rate of increase in penile length and testosterone in the injection group compared to the pump group. However, significant increases in testicular volume, AMH, inhibin B, and FSH concentrations, as well as testicular descent, were observed with either central hormone replacement modality. The study was a retrospective comparison; no long-term outcomes were reported.

**Figure 6.**
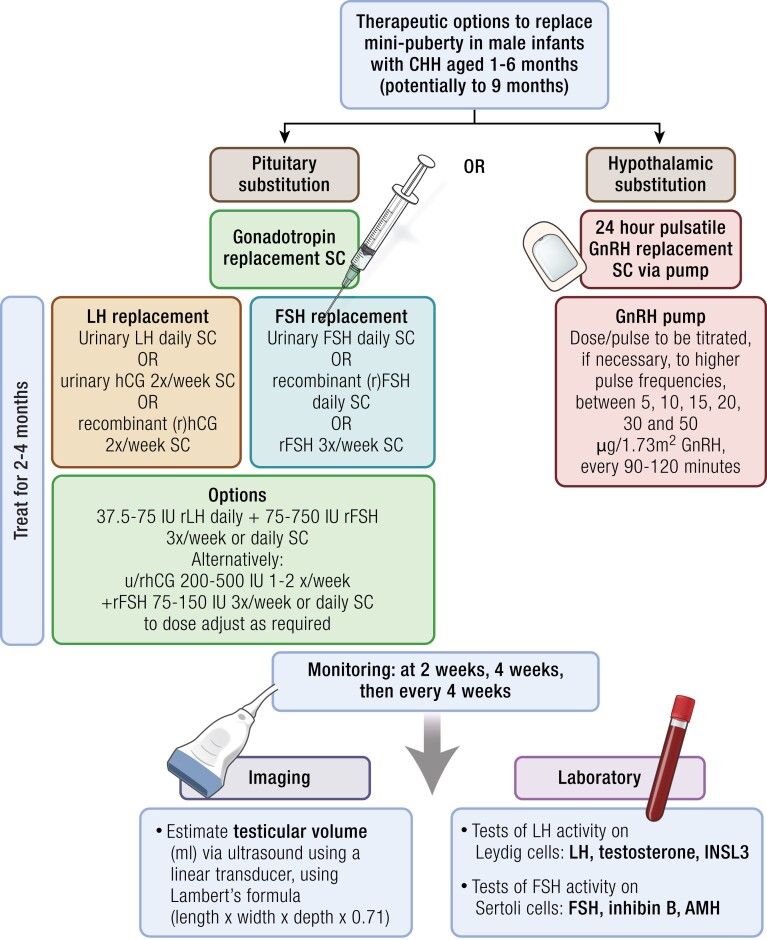
Therapeutic options for central hormone replacement during mini-puberty for male infants with suspected congenital hypogonadotropic hypogonadism (CHH), and recommended monitoring.

At present, gonadotropin therapy during the first 6 months of life is not yet formally recommended as a routine therapy for the induction of mini-puberty in GnRH deficiency. The various possible approaches to replacement of mini-puberty are summarized in Fig. 6.

**Our consensus view is that the most practical and appropriate approach would be to perform daily injections of recombinant LH and rFSH, which are available in a fixed 1:2 combination in a pen for a treatment course of 4-5 months, with the commencement of treatment during the period of physiologic mini-puberty, that is, before age 6 to 9 months. A suggested approach is to commence with a low dose, for instance, 37.5 IU recombinant LH and 75 IU rFSH per day. This dose can then be increased 1 or 2 weeks later, if serum LH concentrations do not rise into the target physiological range. Supraphysiological FSH serum concentrations that are likely to occur with such a regimen are not likely to be detrimental, due to the localization of the FSHR only on Sertoli cells and due to observations for example in moderate preterm mini-puberty, when such enhanced stimulation occurs without side effects.** While replacement of minipuberty can be performed using either pulsatile GnRH or gonadotropins male infants with isolated CHH, replacement of CHH in boys with combined pituitary hormone deficiencies (CPHD) will be successful only with the use of gonadotropins.

After therapy is commenced, monitoring should take place at regular intervals, such as at 2 weeks, 4 weeks, then every 4 weeks, post commencement. Monitoring should encompass imaging, including measurement of testicular volume and blood tests targeted to LH and FSH activity.

However, despite the lack of formal recommendation for routine use of gonadotropins for the treatment of cryptorchidism, gonadotropin therapy was administered for 23% of infants with UDT of undetermined origin in one survey conducted in Italy (314). The Consensus Statement of the European consortium studying GnRH biology described gonadotropins as possibly beneficial for additional stimulatory effect on gonadal development for infants aged 1 to 6 months, but noted that further studies are needed to evaluate gonadotropins and quantify long-term outcomes. The comprehensive Endo-European Reference Network 2022 Guideline on pubertal induction in patients with congenital gonadotropin deficiency did not recommend gonadotropins for the induction of mini-puberty (316).

The initiation of gonadotropins for induction of mini-puberty is therefore at present a decision to be made by specialists in pediatric endocrinology, with appropriate counseling for parents or carers regarding the potential known short-term outcomes, adverse effects, and uncertain long-term effects.

## Key Outstanding Questions

### Possible Side Effects of Gonadotropin Use in Infants and Evidence for Insufficient Efficacy

#### Local side effects

Likely side effects may be related to route of administration, such as pain and skin irritation from subcutaneous injections (317). Insulin pump devices that have been repurposed for continuous subcutaneous infusions of gonadotropins have reported side effects such as scars, lipohypertrophic areas, and in rare cases minor abscesses or blisters (318).

#### Human chorionic gonadotropin antibodies

Reported adverse effects from gonadotropins have included the development of antibodies to hCG after treatment in one case, which was successfully treated by switching to LH and FSH (319).

#### Gynecomastia

Breast development and increases in estradiol have also been reported (320). However, this occurs primarily if supraphysiologic doses of hCG are used in adolescents or adults with CHH, as these will cause enhanced serum testosterone concentrations that in turn give rise to aromatization to estrogens in adipose tissues.

#### Spontaneous erections

An increased frequency of spontaneous erection may be observed during this treatment, as may be seen with testosterone or DHT therapy.

#### Other side effects

Among the studies in mini-puberty shown in Table 5, adverse effects were reported by some studies, but without control groups it is challenging to determine causation. One patient treated with LH and FSH injections developed a slight increase in body hair and pigmentation, a local rash, sleep disturbance, and otitis media, but no serious adverse effects (306). In five cases testicular reascent post treatment were reported. These were then treated with orchidopexy (176, 228, 315). Other studies reported no adverse effects (228, 307, 308).

#### Theoretical considerations regarding potential side effects

As noted, the absence of testicular Sertoli cell androgen receptor expression in infancy is the reason why meiotic divisions of spermatogonia and thus activation of spermatogenesis is not likely to occur in the male infant on GnRH or combined LH (or hCG) + FSH substitution.

A key theoretical adverse effect, which has not been studied in detail in infants with hypogonadotropic hypogonadism, is the potential effect of hCG treatment on the germ cell population. A study of the administration of hCG in prepubertal rats showed that the subgroup treated with high-dose hCG had a significantly reduced germ cell haploid cell population compared to untreated and lower-dose groups (321). In humans, increased apoptosis of spermatogonia was demonstrated in adult patients treated with hCG and orchidopexy, compared to orchidopexy alone (322). In 1 study of boys aged 1 to 3 years with cryptorchidism, patients treated unsuccessfully with GnRH or hCG prior to orchidopexy had a lower number of spermatogonia per tubule in testicular biosies than those who underwent surgery alone (323). However, other studies have reported patients receiving gonadotropin therapy as more likely to have normal histology of spermatogonia, and those treated with hCG as having significantly greater numbers of spermatogonia than untreated controls even at 6 to 9 months post treatment (324, 325). The mechanism behind the possible role of hCG on inducing apoptosis of spermatogonia is unknown, with one suggestion that it may be linked to a rapid fall in testosterone after cessation of treatment (298). However, it should be noted that these studies have been conducted in broad populations with cryptorchidism, rather than in CHH specifically. Overall, the potential effect of hCG in this domain for patients with hypogonadotropic hypogonadism is uncertain, and hCG monotherapy should be used with caution. Large, prospective, long-term, and randomized studies are needed to more accurately quantify potential adverse effects and complexities associated with treatment.

## Midterm Outcomes After Physiological Replacement of Mini-Puberty

Thus far, 2 studies have conducted midterm follow-up. No data on long-term follow-up during pubertal induction in adolescence have been published so far in males previously treated with central hormone replacement in infancy.

In the REMAP study (REplacement of MAle mini-Puberty), Papadimitriou et al described the administration of daily injections of Pergoveris (LH/FSH 75/150 IU) for 3 months to 10 infants, median age 4.2 months, with micropenis and/or cryptorchidism due to CHH (315). In the short term, this therapy led to increased LH, FSH, inhibin B, AMH, and testosterone to within or above normal concentrations, as well as increased stretched penile length and testicular descent in all patients, measuring an average of 1.5 mL. After 3 to 10 years of follow-up, all testes remained in the scrotal position, although a regression in testicular size to an average of 1.0 mL was observed. Mild regression in size of the testes after mini-puberty is a phenomenon also noted in healthy children (35).

The second mid-term follow-up study, conducted by Kohva et al (176), described administration of FSH injections twice or thrice weekly for 3 to 4 months combined with a once-monthly injection of testosterone for 3 months to 5 infants with CHH, starting at a mean age of 2.5 months. In the short term, this led to a significant increase in inhibin B and penile length, and some increase in testicular volume although 2 patients had testicular reascent post treatment. After 11.2 ± 1.5 years, for 3 boys for whom results were available, inhibin B concentrations did not differ from untreated controls. However, penile length and testicular volumes were not reported at this stage, and compared to other studies shown in Table 5, the administration of gonadotropins was relatively restricted; target serum FSH concentrations were not achieved in the majority of patients during treatment in this study (176). Other studies all described administration of FSH in combination with LH and administered it more frequently and at higher doses.

## Research Priorities for Further Study of Mini-Puberty

Despite encouraging short-term outcomes, there is an absence both of controlled clinical studies and of studies assessing long-term outcomes, including later spermatogenesis and fertility, which at present can only be inferred or presumed from observed increases in testicular volumes after induced mini-puberty. Studies have also been performed in small numbers of cases, precluding detailed statistical analyses. There is also a lack of studies comparing different regimens (326).

Most studies investigating male mini-puberty have administered gonadotropins at or after around age 4 to 6 months, when physiological mini-puberty is usually complete. It is unknown whether there may be any negative consequences from a delayed induction of mini-puberty, and thus whether accelerated early diagnostics will be important for fertility preservation.

Optimal length of treatment with central hormone replacement has not been investigated yet. Comparison of different regimens and ages, and follow-up into adulthood, would be recommended for future studies to quantify the effect on spermatogenesis or fertility outcomes (91), with additional monitoring of psychological well-being (157, 288).

Characterization of mini-puberty and its significance in female patients is also needed, which is a challenge due to the absence of “red flag” features that may raise suspicion of CHH (169). It is also less urgent, as female patients respond well, and certainly better than males, to pharmacological for induction of fertility (120). Nevertheless, early identification and diagnosis may help alleviate negative psychological outcomes for these patients (253).

Another area deserving further research relates to the interrelation of reproductive and neurocognitive pathways. Recent work has raised the potential effect of disordered mini-puberty on cognition. A mouse model of Down syndrome showed that progressive postpubertal sensory and cognitive deterioration was seen in association with an age-dependent decline in GnRH production, associated with decreased expression of *GnRH1* and imbalances in transcription during mini-puberty (327). Interestingly, administration of pulsatile GnRH reversed cognitive decline both in a mouse model and in a pilot study of 7 men with Down syndrome. Patients with Down syndrome have an increased risk of cryptorchidism, due to both central and primary gonadal defects. This study suggests a primary role for GnRH deficiency in this condition. This raises the question of whether shared reproductive and neurocognitive phenotypes are caused by common neural development pathologies in different neuronal populations, or due to common deficiency of GnRH-specific pathways (328).

The wider health implications of disordered mini-puberty, in addition to testicular development and fertility in males, should also be characterized. Specifically, the mechanisms by which mini-puberty may have broad metabolic, cardiovascular, reproductive, neurological, and behavioral consequences could help maximize long-term outcomes for these patients (329).

**In summary, there is an urgent need to collect prospective data on outcomes and side effects from treatment with gonadotropins both in infants and adolescents. This could be achieved by data collection within the existing I-DSD registry (330), which is a prospective international registry containing data from 86 centers on patients with differences of sex development and maturation. Use of this registry could also increase clinician confidence and collaboration for treating to mimic mini-puberty during the first months of life.**

## Conclusions

Disordered mini-puberty in the context of CHH, although rare, has wide-reaching and significant consequences for patients in terms of secondary sexual development, fertility, and psychological sequelae. There is a clear impetus toward an early diagnosis of male patients in infancy, particularly in the presence of red flags such as cryptorchidism and micropenis. Gonadotropins have been used successfully in small studies for the replication of male mini-puberty, leading to positive short-term markers of improved testicular function and to descent of maldescended testes. There is an evidence gap regarding the significance of female mini-puberty.

Although large, long-term, prospective, and randomized studies would be the gold standard to answer remaining questions, this is impeded by the rarity of the condition. Therefore, a more pragmatic observational approach that promotes the collection of data of individual treatment outcomes and observed side effects of central hormone replacement strategies in an international registry seems to be reasonable. It is hoped that this could help unravel the mysteries around the significance of mini-puberty, the optimal regimens, and long-term benefits of central hormone replacement in male infants.

## Abbreviations

ACTH: adrenocorticotropin
AMH: antimüllerian hormone
BBS: (Laurence-Moon-) Bardet-Biedl syndrome
BMD: bone mineral density
CHH: congenital hypogonadotropic hypogonadism
CPHD: combined pituitary hormone deficiency
DHT: 5-dihydrotestosterone
DSDs: differences of sexual development
EDCs: endocrine-disrupting chemicals
FSH: follicle-stimulating hormone
GnRH: gonadotropin-releasing hormone
hCG: human chorionic gonadotropin
HPG: hypothalamic-pituitary-gonadal
INSL3: insulin-like factor 3
LH: luteinizing hormone
MRI: magnetic resonance imaging
PSAI: Pre-School Activities Inventory
PSIS: pituitary stalk interruption syndrome
rFSH: recombinant follicle-stimulating hormone
TSH: thyroid stimulating hormone (or thyrotropin)
UDT: undescended testes
